# A Dynamic Graph–Based Multiobjective Optimization Method for Physician Recommendation: Development and Evaluation Study

**DOI:** 10.2196/88854

**Published:** 2026-07-31

**Authors:** Shuang Geng, Rui Wang, Wenli Zhang, Ben Niu

**Affiliations:** 1 Deparment of Management Science, College of Management, Shenzhen University, 1066 Xueyuan Road, Shenzhen, 518000, China, 86 852 2653 4283; 2 Ivy College of Business, Iowa State University, Ames, IA, United States

**Keywords:** physician recommender system, multiobjective optimization, dynamic physician graph, data sparsity, online health care

## Abstract

**Background:**

Online health care consultation provides patients with broad access to physicians, but also presents the challenge of selecting a suitable doctor in the absence of triage guidance. Patients have multifaceted needs, prioritizing not only recommendation accuracy but also physician service quality, and diversity of physician expertise. Furthermore, the sparsity of patient interaction data intensifies the difficulty of providing balanced and effective recommendations.

**Objective:**

This study aims to develop a multiobjective physician recommendation method that simultaneously optimizes recommendation accuracy, service quality, and diversity of physician expertise while addressing the data sparsity challenge inherent in online health care platforms.

**Methods:**

We propose dynamic graph–based bacteria colony optimization for multiobjective physician recommendation (DyGMO-PR), a dynamic graph–based multiobjective optimization method. It integrates bacterial colony optimization with an evolving physician relationship graph. Our approach features a novel graph–based encoding scheme, a chemotaxis-inspired graph-walking strategy for stable search, and a dynamic graph evolution mechanism that learns implicit physician relationships to enhance recommendation quality under sparse data conditions.

**Results:**

Evaluation on a real-world dataset comprising 10,493 consultation records from 1256 patients and 1377 physicians demonstrates the effectiveness of DyGMO-PR. Our method consistently outperforms 6 state-of-the-art multiobjective recommendation algorithms. Using a recommendation list length of 6 as an example, DyGMO-PR achieves an accuracy of 0.876, a service quality of 0.873, and a diversity of 0.720, surpassing the best-performing baseline by 7.4%, 3.1%, and 8.1%, respectively. DyGMO-PR also attains the highest hypervolume value (0.696 at *K*=6) and the highest recall (0.380 at *K*=6). Case studies and graph analysis further demonstrate the method’s effectiveness in generating interpretable and balanced recommendation lists.

**Conclusions:**

DyGMO-PR offers an effective solution for multiobjective physician recommendation. It provides a flexible and practical foundation for building more responsive and reliable recommender systems in online health care.

## Introduction

Online health care consultation services have emerged as one of the most promising solutions for mitigating health disparities and improving access to health care services [1,2]. Notably, China, one of the global leaders in online health care, has upheld it as a national health priority to reduce health care costs and improve access in rural areas [3]. In the United States, investors view online health care consultation as the new backbone of the future health care system [4]. The European Union has also embraced online health care, integrating it into its Regional Digital Health Action Plan [5].

One notable feature of online health care consultation is its ability to overcome geographical and time constraints, which encourages more medical professionals to provide services online [6]. For instance, HaoDaiFu, one of China’s largest online health platforms, provides access to over 910,000 physicians. Another feature of online health care consultations is that patients typically do not have access to triage services commonly available in offline health care settings. This challenge is further compounded in certain countries and regions (eg, China) where patients do not have a primary care physician and must directly choose a specialist. For individuals with limited medical knowledge, even after identifying the appropriate type of specialist, the sheer number of available physicians remains overwhelming [7]. Furthermore, patients may have diverse preferences and expectations. Without an effective matching mechanism, discrepancies between patient expectations and the services provided by physicians can lead to dissatisfaction, delay timely treatment, and adversely affect the overall reputation of online health care platforms [8,9].

Physician recommender systems are crucial techniques that help patients receive personalized physician recommendations that align with their preferences [10]. Especially in the online context, these systems play a crucial role in reducing the time and effort patients spend searching for physicians [11]. They are essential for improving patient satisfaction, clinical outcomes, and overall efficiency within online health care platforms [12].

**Figure 1.**
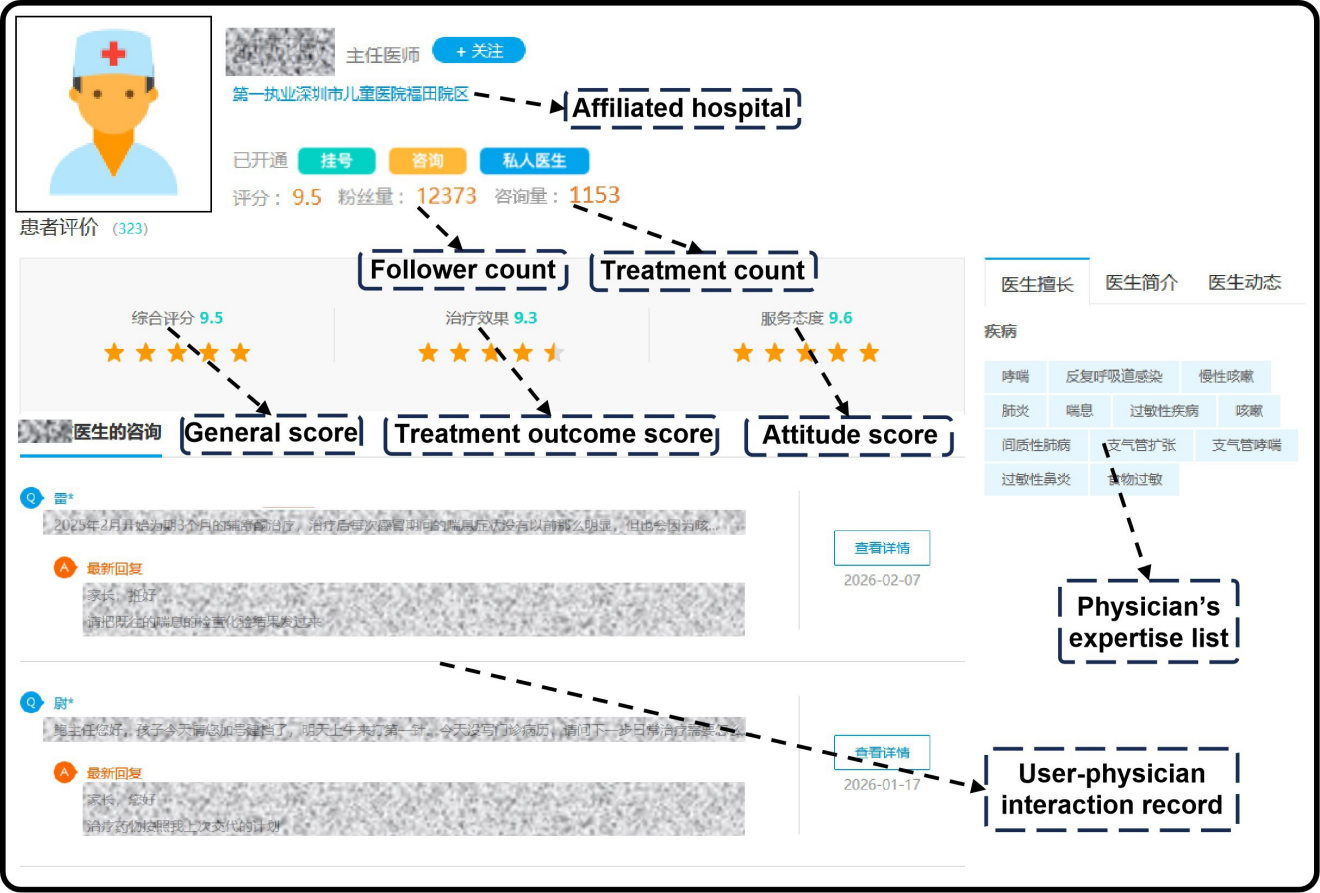
Physician information from an online consultation platform [14].

Most existing physician recommender systems learn from patients’ explicit descriptions and historical consultation records to perform patient-physician matching [13]. However, they often fail to account for the full complexity of patient preferences. In the online context, patients’ needs extend beyond merely identifying a physician with relevant expertise. As depicted in Figure 1 [14], physician information displayed in an online consultation platform is multifaceted, and all this information collectively influences patients’ selection choices. Therefore, besides the recommendation accuracy goal in conventional system design, we integrate 2 more objectives in our recommendation framework, including physician service quality (derived from the physician’s attitude score, treatment outcome score, affiliated hospital tier level, average reply time, etc) and expertise diversity (derived from the physician’s expertise list, estimating the physician’s capability in tackling patients’ multidisciplinary inquiries). The diversity of physician expertise is particularly valuable when patients are uncertain about their condition or lack clear medical knowledge [15]. It should be noted that these objectives may conflict with one another, making it challenging to simultaneously satisfy all of them in a single recommendation list.

Early physician recommender systems primarily focused on improving recommendation accuracy as a single objective. They typically rely on historical patient-physician interaction data, using collaborative filtering or deep learning models to estimate the alignment between physicians and patient needs. For instance, Zheng et al [16] and Lu et al [17] combined Bidirectional Encoder Representations from Transformers (BERT) with attention mechanisms to learn representation vectors from patient-physician dialogues and profiles. Then they generate recommendations by computing matching scores. To alleviate the cold-start problem caused by data sparsity, Mondal et al [18] constructed a multilayer graph database to model patient-physician trust relationships. Zhang and Li [13] integrated medical knowledge graphs with deep networks to enhance recommendation reliability. Although these studies have effectively improved accuracy, they remain limited to single-objective optimization. These accuracy-oriented studies fail to address the multidimensional needs of patients in online health care consultation scenarios.

In recent years, some researchers have begun to explore multiobjective physician recommendation in online health care settings. Wang et al [19] considered recommendation accuracy and hospital diversity as optimization objectives. They use matrix factorization to estimate physician competency and use heuristic algorithms to enhance diversity during the ranking stage. Fei and Li [20] used the entropy-weighted method to fuse symptom matching, professional qualifications, patient preferences, and resource equity into a single score for ranking in their system design. Zhao et al [21] maximized patient satisfaction and physician disease-type preferences by adopting linear weighting to convert the multiobjective problem into a single-objective model. Cui et al [22] estimated the uncertainty of matching scores and ranked physicians based on the lower bounds of confidence intervals, thereby indirectly balancing accuracy and risk. Although these studies have broadened the objective dimensions of physician recommendation, they still convert multiobjective problems into a single composite indicator or ranking and therefore fail to capture the inherent trade-offs among conflicting objectives.

Multiobjective evolutionary algorithms can generate a Pareto front that offers decision makers a set of nondominated solutions that balance multiple conflicting objectives [23,24]. These algorithms have been successfully applied in recommender systems. For instance, Jain et al [25] enhanced nondominated sorting genetic algorithm II (NSGA-II) for collaborative filtering, and Geng et al [26] developed a trajectory-reinforced bacterial colony optimization (BCO) approach for multiobjective recommendation. However, their application to online physician recommendations remains limited. A significant challenge of physician recommender system design in an online consultation context is the inherent sparsity of patient health records. Since online consultations typically serve as a supplement to in-person health care, they represent only a small fraction of a patient’s medical history [27]. Thus, the historical patient-physician interactions only reveal partial patient preferences. The sparse solution space may lead to poor convergence and suboptimal performance. How to achieve multiobjective recommendations that balance patients’ diverse needs under such sparse data conditions thus remains an underexplored research challenge.

To address the research gap identified above, this study seeks to answer the following research question (RQ):

RQ1: How can a multiobjective optimization method be developed to simultaneously optimize recommendation accuracy, physician service quality, and diversity of physician expertise?

RQ2: How to develop a graph-based optimization framework that mitigates data sparsity in patient-physician interactions and provides interpretable guidance for decision-making?

To address these challenges, this study proposes a novel approach named dynamic graph–based bacteria colony optimization for multiobjective physician recommendation (DyGMO-PR). The main contributions of this study are 2-fold.

First, we introduce a novel multiobjective optimization method for physician recommendation for online health care consultations. This method leverages an evolutionary algorithm specifically designed to address the issue of sparse patient data in online health care consultations. The objective is to achieve an optimal balance among 3 critical goals, including recommendation accuracy, physician service quality, and the diversity of physician specialties in the recommendation list. The strength of our approach lies in its ability to incorporate multiple criteria, enabling a more balanced and comprehensive physician recommendation process.

Second, within this method (1) we develop a physician relationship graph-based encoding technique that effectively captures implicit relationships between physicians, thereby enhancing recommendation quality even with limited patient data. This approach addresses the challenge of sparse patient records; (2) we propose a graph walking–based search and graph evolving method to improve the stability of the heuristic optimization process. This method dynamically updates the physician graph structure, allowing the system to evolve and adapt, resulting in enhanced recommendation performance; and (3) by integrating these techniques with a multiobjective bacteria colony optimization algorithm, we create a novel multiobjective recommendation system that offers a new approach to managing the complexities of sparse patient data and patients’ multidimensional needs.

Extensive experiments on a real-world physician recommendation dataset are conducted to evaluate the performance of our proposed method. The results demonstrate that our method outperforms existing state-of-the-art methods. Furthermore, we conducted case studies to validate the effectiveness of the proposed method in improving recommendation performance and enhancing explainability, thereby providing actionable insights for both patients and online consultation platforms.

## Methods

### Problem Formulation

We formalize the physician recommendation problem as a discrete multiobjective optimization problem. Let $U=\{u_{1},u_{2},...,u_{M}\}$ denote the set of patients on an online health care consultation platform, and $P=\{p_{1},p_{2},...,p_{N}\}$ denote the set of physicians. For each patient *u* ∈ *U*, we have the physicians they previously selected on the platform, denoted by a list of physicians $P_{u}=\left\{p_{u}^{1},\;p_{u}^{2},\;\ldots ,\;p_{u}^{l}\right\}$. Additionally, each physician is associated with a set of diseases they specialize in treating $S=\left\{s_{1},s_{2},...,s_{D}\right\}$, a hospital tier level *q^hospital^*, an average physician service score *q^score^*, and the number of endorsements (thumbs-up) received from colleagues *q*^*colleague*^. Our objective is to generate a list of physicians for a target patient *u* that achieves Pareto-optimal performance across 3 key objectives, namely prediction accuracy, physician service quality, and diversity in the diseases treated by the physicians. The formal definition of the recommendation problem and Pareto dominance rule are provided below.

#### Definition 1

The recommendation problem with 3 objectives and *K* decision variables is given by

minimize $F(x)=\{f_{1}(x),f_{2}(x),f_{3}(x)\}$

where *x* is the decision vector and *F (x)* is the objective vector.

#### Definition 2

Let *x_A_* and *x_B_* denote 2 decision vectors in the solution space. *x_A_* dominates *x_B_* if and only if


$$\begin{array}{c} \forall_{i}=1,2,3:f_{i}(x_{A})\leq f_{i}(x_{B})\\ \exists_{i}=1,2,3:f_{i}(x_{A})§lt;f_{i}(x_{B}) \end{array}$$

The Pareto optimal front consists of nondominated decision vectors in the solution space. For each solution on the Pareto front, no objective can be further improved without compromising others. Therefore, the Pareto front provides a set of optimal solutions, each offering a different trade-off between the 3 objectives, thus presenting a wide range of performance options for the physician recommendation task.

### Optimization Objectives

This work simultaneously optimizes recommendation accuracy, physician service quality, and diversity of physicians’ areas of expertise. It is important to note that these different objectives can vary depending on the specific recommendation system. In the context of patients selecting physicians on an online health care consultation platform, we defined our 3 objectives based on the goals that patients commonly consider. Moreover, these 3 objectives often conflict with one another.

The accuracy objective ensures the accuracy of recommendations. It uses the widely recognized metric for recommendation systems [28], which compares the recommendation results with patients’ ratings while accounting for both the relevance and ranking position of physicians:


(1) $$DCG=\sum_{i=1}^{\left|K\right|}\frac{r_{u,i}}{\log_{2}(i+1)}$$

(2) $$NDCG=\frac{DCG}{IDCG}$$

where *K* represents the number of physicians included in the recommendation list, and *r_u,i_* represents the predicted rating of *i^th^* physician by user *u*. Ideal discounted cumulative gain (IDCG) represents the ideal rating of discounted cumulative gain (DCG), which reflects the highest value when the most relevant physicians are ranked at the top. The *r_u,i_* is defined based on patients’ historical consultations. Specifically, for physicians with whom a user has interacted, we set *r_u,i_*=1 whereas for unrated physicians, we used extremely simple graph contrastive learning (XSimGCL) [29] to estimate the missing ratings. In our work, XSimGCL is trained using the historical interactions between users and physicians. XSimGCL is a popular contrastive learning method for recommendation. It has demonstrated superior performance in both recommendation accuracy and training efficiency, particularly on highly sparse datasets.

Given similarly accurate recommendations, patients generally prefer to choose doctors who offer higher-quality services. The measurement of physician service quality is a composite index that incorporates the physician service scores, the tier level of the physician’s affiliated hospital, and endorsements from colleagues.

Physician service scores include the average reply time score, attitude score, treatment quality score, productivity score, and comment score, all of which are recorded by online health care consultation platforms.

The tier level of the physician’s affiliated hospital is used to reflect service quality because our data source is from a platform based in China, where the hospital’s tier generally indicates the physician’s level of expertise. Physicians in higher-tier hospitals are often associated with higher service quality. In China, hospitals are classified into 4 tiers (0-3), with level 3 representing the highest service quality.

The endorsements (thumbs-up) from colleagues reflect the outcomes of peer reviews and can indirectly indicate the quality of a physician’s services.

For a recommendation list with *K* physicians, we design a score to calculate the physician service quality:


(3) $$Quality=\frac{1}{\left|K\right|}(\sum_{i=1}^{\left|K\right|}\left(q_{i}^{hospital}+q_{i}^{score}+q_{i}^{colleague}\right))$$

where *q_i_*^*hospital*^, *q*_*i*_^*score*^, and *q_i_*^*colleague*^ are normalized. This comprehensive measure provides a good representation of a physician’s service quality.

If the accuracy of recommendations and the quality of the physicians’ services are comparable, the diversity of diseases in which the recommended physicians specialize can further enhance the likelihood of successful recommendations. In our research context, patients, as nonexperts, must independently select their physician without the assistance of triage or referral services. When presenting a list of recommendations, ensuring that the physicians are relevant to the patient’s condition while also maximizing the breadth of diseases they are skilled at treating can significantly enhance the patient’s ability to choose from a broader range of options. This, in turn, increases the likelihood of a successful recommendation.

For a recommendation list with *K* physicians, the diversity of physician expertise is measured as


(4) $$Disease\_diversity=\frac{\left|s_{K}\right|}{c*\left|s_{top\_l}\right|}$$

where |^*S*^*K*| represents the total number of diseases treated by the recommended *K* physicians, |*^S^top_l*| denotes the total number of diseases treated by the top *l* most skilled physicians, which is used to normalize the diversity measure, and *c* is a constant that sets the range of diversity measure.

### Model Development

We propose a novel approach, DyGMO-PR, which incorporates several key algorithmic innovations. BCO [30] is a bio-inspired algorithm known for its strong global search capability and fast convergence in multiobjective tasks [26,31,32]. Our method introduces a multiobjective optimization framework based on BCO that simultaneously balances recommendation accuracy, physician service quality, and the diversity of physician expertise in the recommendation results. Within this framework, we present two novel algorithmic components that distinguish our approach from prior studies: (1) We construct a physician graph and propose a new graph-based encoding and updating method for physician recommendation lists. This technique enhances the stability of both heuristic and stochastic optimization processes, leading to more robust recommendation outcomes. Furthermore, this encoding strategy serves as the foundation for the subsequent dynamic graph search. (2) We develop a dynamic graph evolution mechanism that adaptively refines the physician graph structure by uncovering implicit relationships among physicians. This enables the identification of high-quality recommendation candidates beyond those explicitly known to the system.

**Figure 2.**
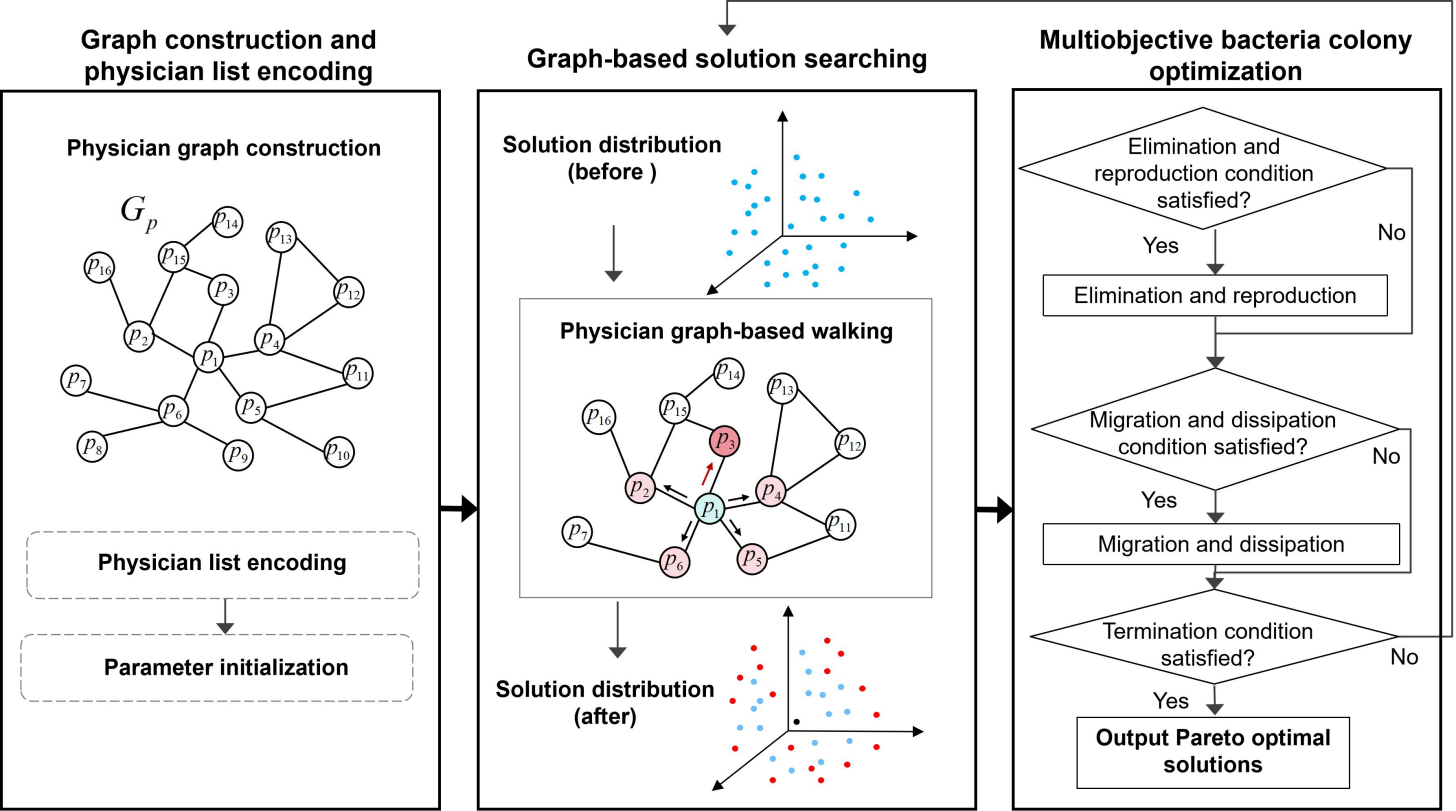
The dynamic graph–based bacteria colony optimization for multiobjective physician recommendation (DyGMO-PR) framework; P: physician.

The details of the research design are elaborated below. Figure 2 provides an overview of the proposed DyGMO-PR framework.

#### Physician Graph Construction and Physician List Encoding

DyGMO-PR first constructs a physician graph to represent the relationships between physicians and proposes a novel method for encoding physician lists, where each list serves as a candidate for physician recommendation.

The physician graph, *G_p_*=(*P,E*), is defined as follows: each node *P* represents a physician, and an edge *e* exists between 2 physicians if they share expertise in treating at least one disease. The graph *G*_*p*_ captures relationships in physicians’ areas of medical expertise, and the edges serve as heuristic pathways for identifying candidate physicians.

**Figure 3.**
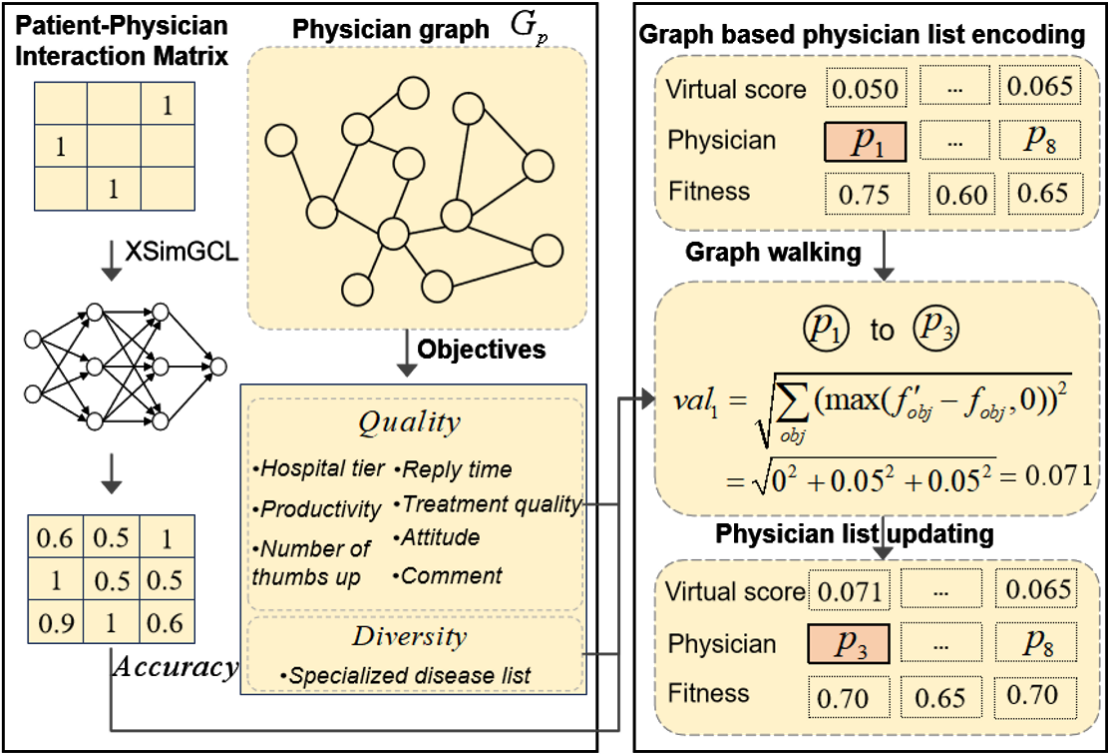
Graph-based physician list encoding and updating. P: physician; XSimGCL: extremely simple graph contrastive learning.


Based on *G*_*p*_, we propose a new physician list coding scheme in the bacteria colony algorithm, as illustrated in Figure 3. Each bacterium *bacteria_u,j_* (*j^th^* bacteria for user *u*) represents a physician recommendation list for recommendation results. The coding of *bacteria*_*u,j*_ contains three key elements: (1) a list of recommended physicians {*p*_1_, ...,*p_K_*}, (2) a set of virtual scores measuring individual physician contributions to recommendation objectives {*val*_1_, ...,*val_K_*}, and (3) fitness values corresponding to the 3 recommendation objectives: *Accuracy_u_*_,_*_j_*, *Quality_u_*_,_*_j_*, and *Diversity_u_*_,_*_j_*. The fitness values are computed using Equations 1-4. A physician’s virtual score quantifies their contribution to the overall fitness of the bacterium (see Equation 5). This virtual score determines the probability (see Equation 6) of their replacement during the bacterial chemotaxis process (see next section).

(5) $$val_{i}=\sqrt{\sum_{obj\in \{accuracy,quality,diversity\}}{\left(\max({f^{\prime }}_{obj}-f_{obj},0)\right)}^{2}}$$

(6) $$prob_{replace}=\frac{1-val_{i}}{\sum_{i=1}^{K}\left(1-val_{i}\right)}$$

*f*′*_obj_* in Equation 5 represents the fitness value of the current physician list after graph walking to the *i^th^* physician node and *f_obj_* is the fitness value before the graph walking (see the next section) in the previous round of searching. The *prob_replace_* is lower for physicians with higher service quality, better alignment with patient preferences, and greater contribution to the diversity of diseases represented in the physician list. This graph-based coding scheme enables the bacteria colony algorithm to search along the graph paths more effectively and converge more rapidly toward the Pareto front.

#### Physician Graph Walking Inspired by Bacteria Chemotaxis

**Figure 4.**
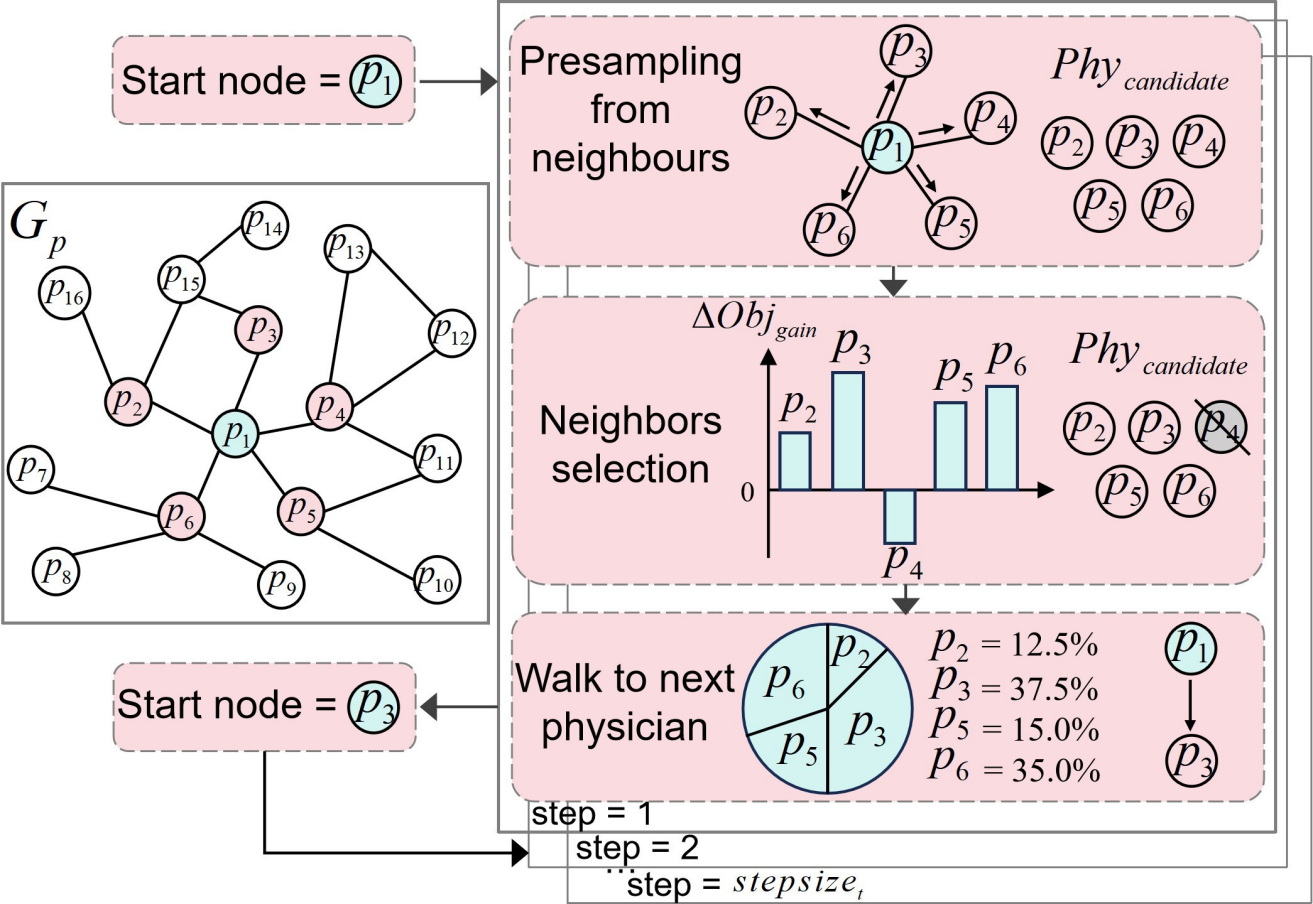
Physician graph walking–based bacteria chemotaxis. P: physician.

The second component (see Figure 4) is a graph-walking technique inspired by bacterial chemotaxis. In graph theory, graph walking refers to traversing a sequence of nodes *P* (ie, physicians), where consecutive nodes are connected by an edge *e*. Our proposed method includes a presampling and node-searching strategy to identify the most suitable physician candidate within the physician graph. This strategy ensures consistent performance improvements during traversal. We further design a physician relationship learning and graph structure evolution strategy. Edges between physicians are added or removed based on their contribution to recommendation performance. The updated graph is then fed back into the first component for subsequent iterations.

Formally, the physician graph *G*_*p*_=(*P*,*E*) defines the solution space for physician recommendation and serves as the foundation for graph walking. For a bacterium *bacteria_u,j_*, a physician *Pi* is selected from its physician list according to the physician’s virtual score (Equation 6). The selected physician becomes the starting point for the graph-walking process. Subsequently, we introduce a neighbor node selection method that leverages presampling. Specifically, a walk step size is set for each random graph walk process. During each random walk, the neighboring physician nodes are included in a candidate list *Phy_candidate_*={*p*_1_, ...,*p_num_*}. The objective gain contributed by each physician on the candidate list is then calculated. Assuming that *p_i_* is replaced by physician *p_can_* in *Phy*_*candidate*_, the objective gain from this replacement is:


(7) $$\Delta obj_{can}=\max\{obj_{can}-obj_{i},0\}$$

(8) $$gain_{can}=\frac{\Delta obj_{can}}{\sum_{can=1}^{\left|Phy_{candidate}\right|}\Delta obj_{can}}$$

where *obj*_*can*_ is the objective function value, randomly chosen from *Accuracy*, *Quality*, and *Diversity*, for the updated physician recommendation list with *p*_*can*_. The larger *gain*_*can*_, the higher the probability that *p*_*can*_ will be selected to update the recommendation list. The algorithm for this graph-walking method, inspired by bacterial chemotaxis, is presented in Figure 4 (the pseudo-code is shown in Multimedia Appendix 1).

For bacterium *bacteria_u_*_,_*_j_*, the chemotaxis process performs a random walk on *G_p_* to update the physicians in its recommendation list. After each random walk, the bacterium updates its virtual score according to Equation 5. The extent of the random walk is controlled by the step size that is initially set as a constant. As the algorithm converges, step size gradually decreases to narrow the exploration scope, thereby enhancing the use of historical exploration information. This approach enables the bacterium to explore a wide range of recommendation combinations during the early iterations, while in later stages, a smaller parameter value helps accelerate the algorithm’s convergence. The step size is updated as below:


(9) $$stepsize_{t}=\left\lceil S_{0}*{\left(1-\frac{t}{T}\right)}^{2}\right\rceil$$

where *t* represents the current iteration number, *stepsize_t_* represents the step size of the iteration *t*, *S*_0_ represents the initial step size, *T* represents the maximum iterations. The final value is calculated by taking the ceiling of the computed result.

After the chemotaxis process, a trajectory reinforcement strategy is used to track the bacteria positions and store the nondominated new solutions using a trajectory document [26]. By merging the bacteria in the trajectory document with the current bacteria positions, the global exploration ability and convergence efficiency of the optimization algorithm are enhanced. The nondominated bacteria positions in the merged population are retained for subsequent operations.

#### Bacteria Elimination, Reproduction, Migration, and Dissipation

The third component involves bacterial elimination, reproduction, migration, and dissipation. These operations help preserve a high-quality bacteria population (ie, high-quality physician lists for the recommendation task) and facilitate the selection of Pareto-optimal outcomes.

Specifically, when the current bacteria population size exceeds a predefined size *Pop*, bacteria with the smallest crowding distances are removed until the population size equals *Pop*. Conversely, if the current bacteria population size is smaller than *Pop*, bacteria with the largest crowding distance will be reproduced to expand the population size to *Pop*. These operations allow the proposed method to search extensively in the solution space in order to find the optimal solution areas.

Formally, a crowding distance measure is used to evaluate the closeness of a bacteria to its neighbors in the 3D objective space:


(10) $$cro\_dis=\sum_{b\in N}\sqrt{\sum_{obj\in \{accuracy,quality,diversity\}}{\left(obj_{b}-obj_{u,j}\right)}^{2}}$$

where *N* contains the 2 nearest neighbor bacteria of *bacteria_u,j_*. Bacteria with smaller crowding distance are located in denser environments and are more likely to be eliminated to maintain population diversity.

#### Dynamic Physician Relationship Learning and Graph Evolving

The last component is designed for dynamic physician relationship learning. We propose a new physician relationship learning and graph evolution mechanism to capture the dynamics of inter-physician relationships.

When constructing the physician graph *G_p_*=(*P*,*E*), an edge *e* is established between 2 physicians if they share common expertise as indicated in their individual profiles. However, this approach may not sufficiently capture the complexity of implicit associations among physicians. For example, a pediatrician and a neurologist are both specialized in treating cephalgia according to their personal profiles, and an edge, *e*, exists between them in the initial physician graph. However, their target patient populations may differ significantly. Therefore, relying solely on the existing edges in *G*_*p*_ for recommendations may limit the framework performance.

To address this, we allow the graph edges in *G*_*p*_ to dynamically evolve and learn valuable node connections throughout the recommendation process. Specifically, let *bacteria*_*u*,*j*_ and *bacteria*_*u*,*j+stepsize*_ represent the bacterium before and after a graph walking, respectively. Each bacterium represents a physician recommendation list. The graph-walking mechanism traverses the physician graph, allowing a bacterium to replace a physician in its current list with an adjacent node. Let *obj_p_accracy_*, *obj*_*p_quality*_, and *obj*_*p_diversity*_ denote the objective gains in accuracy, service quality, and diversity resulting from this graph walk. The graph edge between *bacteria*_*u*,*j*_ and *bacteria*_*u*,*j+stepsize*_ will be updated according to the following rules:

- Rule 1: if *obj*_*p_accracy*_>0, *obj*_*p_quality*_>0, *obj*_*p_diversity*_>0, and no directed edge from *PA* to *PB* exists, this directed edge is added
- Rule 2: if *obj*_*p_accracy*_>0, *obj*_*p_quality*_>0, *obj*_*p_diversity*_>0, and a directed edge from *PA* to *PB* exists, this directed edge is removed
- Rule 3: if there are both positive and negative objective gains among *obj*_*p_accracy*_, *obj*_*p_quality*_, and *obj*_*p_diversity*_, the graph structure remains the same.

It is important to note that these new edge updates are directional. While the initial graph is undirected, the evolutionary process captures asymmetric improvement in objectives when walking from *PA* to *PB*. Therefore, Rule 1 adds a directed edge from *PA* to *PB* to reinforce the productive direction, and Rule 2 removes the directed edge *PB* to eliminate unproductive direction. This design ensures that the evolving graph structure reflects the directional value of physician relationships in the context of multiobjective optimization.

The updating rules refine the physician graph by evaluating whether adding or removing an edge improves the 3 objectives. This process forms the basis for the evolving physician graph *G*_*p*_. As the graph continuously evolves in alignment with the recommendation goal, it becomes increasingly refined and better suited to generating more effective recommendation results.

### Experiment Design

#### Dataset

The dataset used in this study is derived from a real-world online consultation platform. We retain only patients with at least 5 historical consultation records, thereby reducing noise from patients with limited medical knowledge who cannot always choose the most appropriate physician and thus improving the stability and reliability of model training. After this filtering, the final dataset comprises 10,493 online consultation instances, each representing an online physician-patient interaction. The dataset also contains background information about physicians (with private information anonymized), including their service quality scores, areas of disease specialization, professional titles, genders, and affiliated hospitals. These consultation records are organized chronologically. The dataset exhibits a sparsity rate of 99.39%, suggesting a high likelihood of encountering the cold start problem in recommendation tasks and highlighting the challenging nature of physician recommendation in real-world online consultation scenarios.

This study simultaneously optimizes 3 recommendation objectives, including accuracy, service quality, and diversity of physicians’ specialized diseases. Service quality and diversity can be directly computed from physicians’ static information (see Equations 3-4). Accuracy requires the user-physician rating *r_u,i_* (see Euqation 1). For physicians with whom a user has no interaction history, the rating is not directly observable and is predicted by the XSimGCL [28]. To train XSimGCL, we sort each patient’s interaction records chronologically and use the last 20% as the testing set, with the first 80% as the training set. To ensure a valid evaluation, we remove from the testing set any interactions involving physicians who did not appear in the training set. This ensures that all users and physicians in the testing set have been observed during training. The final training set comprises 9178 interactions, and the testing set comprises 1315 interactions. The physician relationship graph is also constructed using the disease specialization information of these 1377 physicians.

#### Benchmark Methods

We compare our proposed method with 6 state-of-the-art multiobjective optimization-based recommendation methods. Below is a brief description of these methods.

- NSGA-II [33]: it uses nondominated sorting and crowding distance to evaluate and select solutions, aiming to provide high-quality Pareto front solutions. It is widely used in various multiobjective optimization problems.
- Multiobjective particle swarm optimization (MOPSO) [34]: it simulates particle movement in the solution space and uses nondominated sorting and an external archive to maintain solution diversity. It is known for its strong global search and convergence capabilities.
- Multiobjective firefly algorithm (MOFA) [35]: it is based on the firefly algorithm, which simulates the light-emitting behavior of fireflies to guide the search process and balances solution quality and diversity by adjusting light intensity.
- Strength Pareto evolutionary algorithm 2 (SPEA2) [36]: it is based on strength Pareto optimization and evaluates solutions using strength metrics and maintains an elite archive of nondominated solutions. Density estimation is used to ensure solution diversity.
- Multiobjective evolutionary algorithm based on decomposition (MOEA/D) [37]: it decomposes the recommendation problem into multiple single-objective subproblems using different weight vectors for trade-offs. It optimizes these subproblems collaboratively, which helps in effectively approximating the Pareto front.
- Trajectory reinforcement-based multiobjective bacterial colony optimization (TRMOBCO) [26]: it proposes an improved multiobjective BCO algorithm that integrates trajectory reinforcement–based chemotaxis, elimination and reproduction based on spatial dependence, and energy-based adaptive migration.

In our evaluations, to ensure fairness, the optimization objectives of these benchmarks are aligned with those of our proposed method, including accuracy, service quality, and diversity in the diseases that physicians specialize in treating within the recommendation results, as defined by Equations 2-4.

#### Performance Measurement

We first evaluate the performance of Pareto-optimal solutions. Following the evaluation metrics commonly used in existing studies, we assess and compare performance based on the mean, SE, and maximum (max) values of the objective functions across the Pareto-optimal solutions.

Additionally, to comprehensively assess the search capability of our method and benchmarks, we adopt the hypervolume metric [38], which measures the volume of the space dominated by the obtained Pareto front relative to a reference point. Hypervolume reflects both the convergence and diversity of the solution set, with a larger value indicating that the algorithm finds a broader and better trade-off among the 3 objectives.

Moreover, in online health care consultation, patients often return to the same physician due to their long-term health care needs and preference for consistent service. Therefore, beyond optimizing the 3 objectives, it is also critical for the recommendation system to retrieve physicians that patients have previously consulted. To assess this capability, we report the Recall@*K* metric, which measures the proportion of historically consulted physicians that appear in the top-*K* recommendation list.

#### Parameter Setting

The parameter settings for the benchmarks are tuned and reported in Multimedia Appendix 2. The length of the recommendation list *K* is set to vary over the range [6,8,10,12,14,16,18,20]. For comparison, the top 50% of solution sets obtained from each method were selected. Each method was executed 5 times, and the average performance was calculated to ensure stability.

### Ethical Considerations

This study was conducted in compliance with relevant national data protection regulations through localized data processing on the source institution’s server, strict anonymization, and access control protocols. The use of online consultation data was approved by the collaborative online consultation service platform and Shenzhen University Ethics Committee (approval number PN-202500098).

## Results

### Overview

Table 1 presents the performance comparison between our proposed method and all benchmarks for the online health care physician recommendation task, evaluated across various recommendation list lengths (*K* values). The bolded values highlight the best results. Among all the comparison methods, DyGMO-PR achieves the highest mean accuracy, mean service quality, and mean diversity, as well as the highest max accuracy, max service quality, and max diversity for these 3 objectives across different values of *K* values. These results indicate that DyGMO-PR consistently generates recommendation results with superior average performance in terms of the 3 recommendation objectives. Furthermore, DyGMO-PR demonstrates a clear advantage over other benchmarks in identifying the best-performing recommendation results.

**Table 1.** Recommendation performance of candidate algorithms with different *K* values.

List length and evaluation metrics	NSGA-II^a^	MOPSO^b^	MOFA^c^	SPEA2^d^	MOEA/D^e^	TRMOBCO^f^	DyGMO-PR^g^
*K*=6							
Accuracy (NDCG^h^)							
Mean (SE)	0.810 (0.097)	0.658 (0.066)	0.726 (0.075)	0.647 (0.053)	0.499 (0.098)	0.816 (0.116)	0.876 (0.144)
Max	0.894	0.743	0.817	0.687	0.603	0.918	0.989
Service quality							
Mean (SE)	0.847 (0.053)	0.774 (0.070)	0.808 (0.054)	0.749 (0.058)	0.783 (0.067)	0.840 (0.066)	0.873 (0.088)
Max	0.890	0.814	0.843	0.778	0.816	0.897	0.946
Diversity							
Mean (SE)	0.666 (0.126)	0.450 (0.086)	0.568 (0.100)	0.429 (0.071)	0.412 (0.069)	0.655 (0.149)	0.720 (0.181)
Max	0.794	0.555	0.675	0.487	0.492	0.808	0.887
*K*=8							
Accuracy (NDCG)							
Mean (SE)	0.806 (0.082)	0.655 (0.058)	0.712 (0.066)	0.645 (0.046)	0.505 (0.094)	0.812 (0.107)	0.875 (0.133)
Max	0.874	0.729	0.786	0.680	0.604	0.899	0.977
Service quality							
Mean (SE)	0.831 (0.043)	0.758 (0.064)	0.786 (0.054)	0.734 (0.053)	0.769 (0.062)	0.824 (0.061)	0.860 (0.077)
Max	0.865	0.796	0.819	0.763	0.800	0.875	0.921
Diversity							
Mean (SE)	0.662 (0.105)	0.448 (0.078)	0.543 (0.092)	0.429 (0.063)	0.413 (0.062)	0.652 (0.137)	0.720 (0.164)
Max	0.767	0.539	0.636	0.481	0.485	0.787	0.867
*K*=10							
Accuracy (NDCG)							
Mean (SE)	0.801 (0.073)	0.654 (0.052)	0.697 (0.059)	0.644 (0.042)	0.512 (0.092)	0.809 (0.100)	0.873 (0.124)
Max	0.858	0.721	0.761	0.676	0.608	0.888	0.965
Service quality							
Mean (SE)	0.817 (0.039)	0.747 (0.060)	0.768 (0.053)	0.722 (0.049)	0.761 (0.058)	0.812 (0.058)	0.848 (0.069)
Max	0.847	0.784	0.800	0.751	0.790	0.858	0.902
Diversity							
Mean (SE)	0.660 (0.094)	0.454 (0.071)	0.522 (0.085)	0.433 (0.059)	0.421 (0.057)	0.654 (0.130)	0.724 (0.152)
Max	0.752	0.535	0.610	0.481	0.487	0.778	0.858
*K*=12							
Accuracy (NDCG)							
Mean (SE)	0.798 (0.068)	0.655 (0.048)	0.687 (0.054)	0.644 (0.038)	0.521 (0.088)	0.805 (0.094)	0.870 (0.115)
Max	0.849	0.718	0.747	0.674	0.613	0.877	0.955
Service quality							
Mean (SE)	0.805 (0.037)	0.737 (0.056)	0.754 (0.052)	0.712 (0.046)	0.751 (0.055)	0.803 (0.056)	0.839 (0.063)
Max	0.832	0.774	0.787	0.740	0.782	0.846	0.888
Diversity							
Mean (SE)	0.674 (0.090)	0.474 (0.069)	0.523 (0.079)	0.450 (0.056)	0.444 (0.056)	0.676 (0.128)	0.748 (0.147)
Max	0.762	0.552	0.608	0.496	0.507	0.798	0.878
*K*=14							
Accuracy (NDCG)							
Mean (SE)	0.792 (0.064)	0.657 (0.045)	0.681 (0.049)	0.650 (0.037)	0.528 (0.085)	0.805 (0.090)	0.869 (0.108)
Max	0.839	0.715	0.735	0.679	0.616	0.871	0.947
Service quality							
Service quality mean	0.795 (0.036)	0.730 (0.053)	0.743 (0.051)	0.706 (0.044)	0.745 (0.052)	0.795 (0.054)	0.831 (0.058)
Max	0.820	0.766	0.777	0.734	0.775	0.836	0.876
Diversity							
Mean (SE)	0.674 (0.086)	0.483 (0.066)	0.521 (0.075)	0.465 (0.054)	0.455 (0.054)	0.686 (0.125)	0.753 (0.138)
Max	0.756	0.558	0.602	0.509	0.516	0.802	0.874
*K*=16							
Accuracy (NDCG)							
Mean (SE)	0.787 (0.060)	0.659 (0.042)	0.678 (0.046)	0.652 (0.034)	0.535 (0.082)	0.800 (0.085)	0.866 (0.101)
Max	0.830	0.714	0.730	0.678	0.622	0.863	0.939
Service quality							
Mean (SE)	0.786 (0.035)	0.724 (0.051)	0.734 (0.049)	0.700 (0.041)	0.738 (0.049)	0.788 (0.053)	0.825 (0.055)
Max	0.811	0.760	0.769	0.726	0.768	0.828	0.867
Diversity							
Mean (SE)	0.697 (0.085)	0.510 (0.066)	0.541 (0.073)	0.490 (0.053)	0.482 (0.054)	0.716 (0.125)	0.785 (0.136)
Max	0.780	0.583	0.621	0.533	0.542	0.830	0.904
*K*=18							
Accuracy (NDCG)							
Mean (SE)	0.784 (0.058)	0.662 (0.040)	0.677 (0.042)	0.653 (0.032)	0.544 (0.080)	0.800 (0.082)	0.863 (0.096)
Max	0.825	0.713	0.724	0.678	0.627	0.859	0.931
Service quality							
Mean (SE)	0.779 (0.034)	0.718 (0.049)	0.727 (0.047)	0.695 (0.040)	0.732 (0.048)	0.783 (0.052)	0.819 (0.052)
Max	0.803	0.754	0.762	0.721	0.763	0.821	0.858
Diversity							
Mean (SE)	0.706 (0.084)	0.526 (0.065)	0.551 (0.071)	0.505 (**0.053**)	0.500 (0.054)	0.730 (0.124)	0.801 (0.133)
Max	0.787	0.597	0.630	0.548	0.557	0.841	0.916
*K*=20							
Accuracy (NDCG)							
Mean (SE)	0.783 (0.056)	0.665 (0.038)	0.676 (0.040)	0.656 (0.031)	0.547 (0.080)	0.799 (0.079)	0.860 (0.091)
Max	0.822	0.714	0.722	0.679	0.631	0.855	0.925
Service quality							
Mean (SE)	0.772 (0.034)	0.713 (0.046)	0.720 (0.046)	0.689 (0.038)	0.727 (0.046)	0.778 (0.051)	0.814 (0.050)
Max	0.796	0.748	0.755	0.714	0.757	0.815	0.851
Diversity							
Mean (SE)	0.671 (0.077)	0.508 (0.060)	0.529 (0.064)	0.487 (0.048)	0.486 (0.050)	0.700 (0.115)	0.765 (0.122)
Max	0.746	0.574	0.599	0.525	0.541	0.803	0.871

a NSGA-II: nondominated sorting genetic algorithm II.

b MOPSO: multiobjective particle swarm optimization.

c MOFA: multiobjective firefly algorithm.

d SPEA-2: strength Pareto evolutionary algorithm 2.

e MOEA/D: multiobjective evolutionary algorithm based on decomposition.

f TRMOBCO: trajectory reinforcement–based multiobjective bacterial colony optimization.

g DyGMO-PR: dynamic graph–based bacteria colony optimization for multiobjective physician recommendation.

h NDCG: normalized discounted cumulative gain.

The SDs of DyGMO-PR are higher than those of some baselines. This allows for broader coverage of the Pareto front and enables the algorithm to offer a more diverse set of high-quality trade-off solutions.

To statistically verify the significance of observed improvements, we performed a one-sided Wilcoxon signed-rank test [39]. Specifically, for a given *K* and a given objective, we compared DyGMO-PR with one baseline at a time using the paired results from the 5 independent runs. The test examined whether DyGMO-PR significantly outperforms the baseline against the possibility that it does not. In all 5 runs, DyGMO-PR outperformed the baseline in every pairwise comparison. Consequently, the sum of positive ranks reached its maximum possible value (W=15), yielding an exact one-sided *P* value of .03. Thus, we reject the null hypothesis at the *P*<.05 significance level, confirming that DyGMO-PR significantly outperforms each baseline in every configuration. Because the result is uniform across all comparisons, we omit individual significance markers in Table 1 for simplicity.

**Figure 5.**
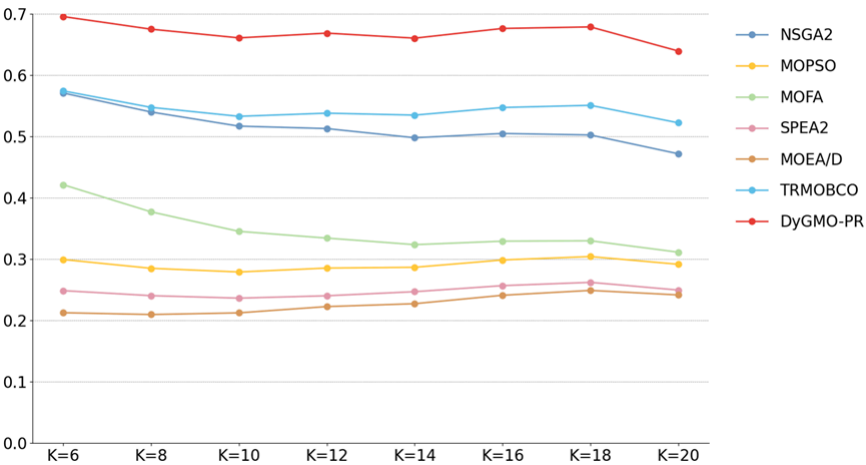
Hypervolume values of compared algorithms. DyGMO-PR: dynamic graph–based bacteria colony optimization for multiobjective physician recommendation; MOEA/D: multiobjective evolutionary algorithm based on decomposition; MOFA: multiobjective firefly algorithm; MOPSO: multiobjective particle swarm optimization; NSGA2: nondominated sorting genetic algorithm II; SPEA2: strength Pareto evolutionary algorithm 2; TRMOBCO: trajectory reinforcement–based multiobjective bacterial colony optimization.

In addition, as shown in Figure 5, DyGMO-PR achieves the highest hypervolume value among the compared methods, demonstrating its capability to balance recommendation accuracy, service quality, and diversity of physician expertise. Its superior performance remains stable across different recommendation list lengths (eg, various *K* values).

Results in Table 2 report the Recall@*K* of compared algorithms. Notably, DyGMO-PR achieves the highest recall among the algorithms. We also conducted the same one-sided Wilcoxon signed-rank test as in Table 1 confirms that DyGMO-PR significantly outperforms all baselines, with *P*<.05 in every case. Additionally, DyGMO-PR also recommends physicians with a wider range of professionalized skills, offering patients a broader choice of options together with their familiar physicians.

**Table 2.** Mean and SD of the Recall@*K* values.

List length	NSGA-II^a^, mean (SD)	MOPSO^b^, mean (SD)	MOFA^c^, mean (SD)	SPEA2^d^, mean (SD)	MOEA/D^e^, mean (SD)	TRMOBCO^f^, mean (SD)	DyGMO-PR^g^, mean (SD)
*K*=6	0.185 (0.151)	0.072 (0.051)	0.193 (0.216)	0.071 (0.144)	0.014 (0.021)	0.278 (0.174)	0.380 (0.150)
*K*=8	0.203 (0.163)	0.076 (0.047)	0.210 (0.215)	0.076 (0.161)	0.013 (0.018)	0.298 (0.167)	0.422 (0.158)
*K*=10	0.198 (0.163)	0.078 (0.049)	0.200 (0.194)	0.076 (0.149)	0.011 (0.017)	0.328 (0.175)	0.453 (0.165)
*K*=12	0.200 (0.154)	0.081 (0.051)	0.179 (0.169)	0.077 (0.149)	0.011 (0.016)	0.338 (0.178)	0.475 (0.169)
*K*=14	0.200 (0.154)	0.082 (0.048)	0.160 (0.147)	0.087 (0.157)	0.011 (0.015)	0.354 (0.179)	0.498 (0.175)
*K*=16	0.196 (0.152)	0.084 (0.049)	0.149 (0.135)	0.089 (0.150)	0.012 (0.018)	0.356 (0.177)	0.513 (0.176)
*K*=18	0.197 (0.143)	0.089 (0.052)	0.142 (0.127)	0.089 (0.157)	0.012 (0.017)	0.370 (0.174)	0.526 (0.179)
*K*=20	0.195 (0.143)	0.091 (0.051)	0.134 (0.113)	0.090 (0.150)	0.012 (0.018)	0.374 (0.178)	0.539 (0.182)

a NSGA-II: nondominated sorting genetic algorithm II.

b MOPSO: multiobjective particle swarm optimization.

c MOFA: multiobjective firefly algorithm.

d SPEA-2: strength Pareto evolutionary algorithm 2.

e MOEA/D: multiobjective evolutionary algorithm based on decomposition.

f TRMOBCO: trajectory reinforcement–based multiobjective bacterial colony optimization.

g DyGMO-PR: dynamic graph–based bacteria colony optimization for multiobjective physician recommendation.

### Sensitivity Analysis

The proposed DyGMO-PR algorithm has 2 important parameters: recommendation list length *K* (which determines the number of recommended physicians displayed to patients) and the initial graph walking step size *S*_0_ (which controls the scope of the physician graph search). We empirically investigate the effectiveness of different parameter settings for these 2 parameters. The value of parameter *K* is chosen from the array [6,8,10,12,14,16,18,20] and *S*_0_ is chosen from the array [2,4,6,8]. Additionally, we also compare the proposed step size decay strategy against using a constant step size to validate the effectiveness of the decay strategy.

**Figure 6.**
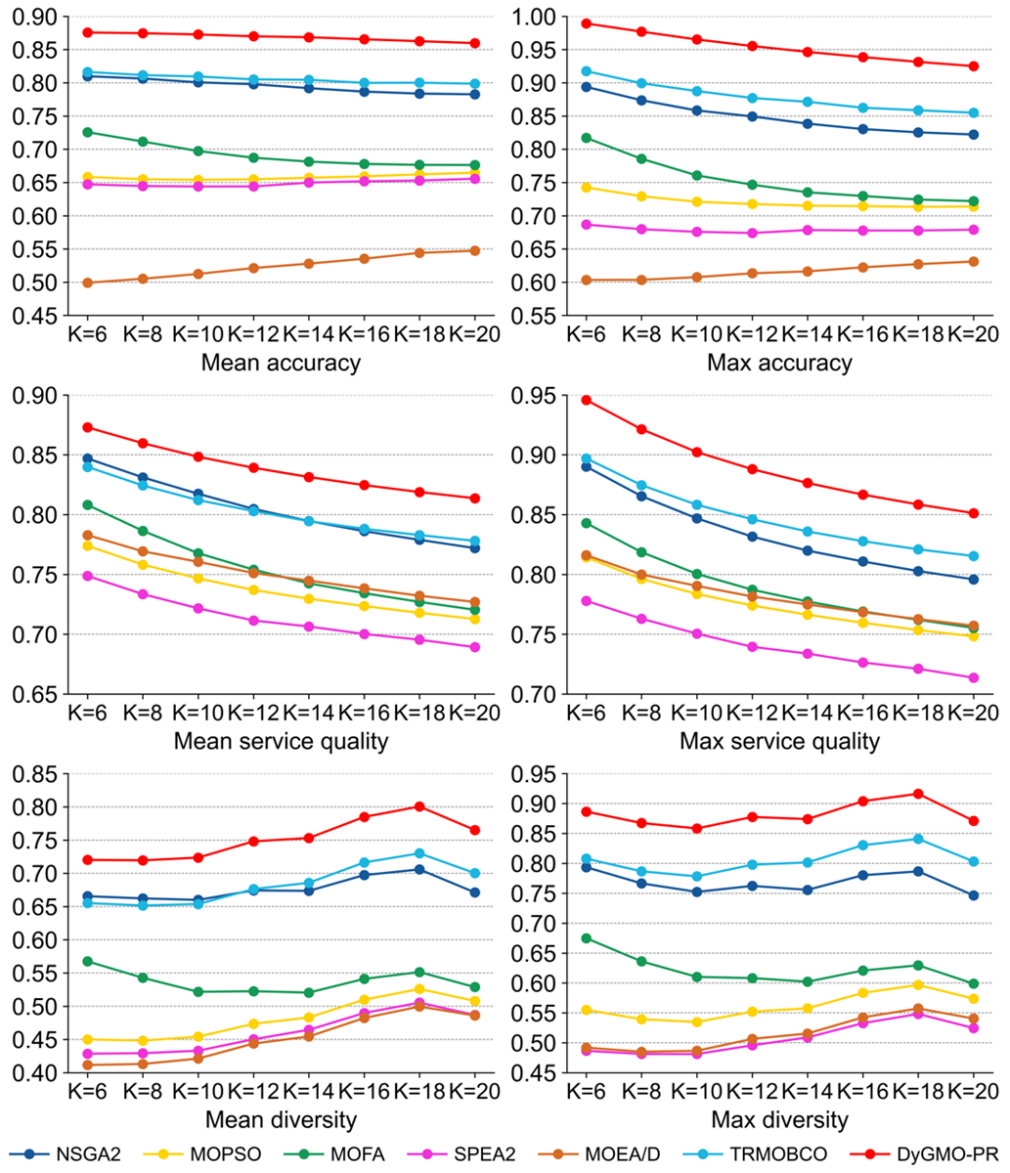
The mean and max performance of compared algorithms. DyGMO-PR: dynamic graph–based bacteria colony optimization for multiobjective physician recommendation; MOEA/D: multiobjective evolutionary algorithm based on decomposition; MOFA: multiobjective firefly algorithm; MOPSO: multiobjective particle swarm optimization; NSGA2: nondominated sorting genetic algorithm II; SPEA2: strength Pareto evolutionary algorithm 2; TRMOBCO: trajectory reinforcement–based multiobjective bacterial colony optimization.

Figure 6 illustrates the performance in terms of mean and max values of the 3 recommendation objectives, including accuracy, service quality, and diversity, respectively. Overall, DyGMO-PR demonstrates a competitive advantage across multiple recommendation objectives and various recommendation list lengths *K*. As the recommendation list length *K* increases, the mean accuracy performance of the algorithms remains relatively stable, while the max accuracy of the algorithms slightly decreases. It suggests that a longer recommendation list may introduce less qualified physicians. In contrast, the mean and max diversity gradually increase as *K* increases until *K*=18, after which the diversity begins to decline with a further increase in *K*. These results indicate that recommending too many physicians to patients is not effective. For patients, the physicians who best match their needs typically appear near the top of the recommendation list. Expanding the list by including more physicians may reduce overall accuracy and service quality, although it can enhance diversity within a reasonably limited list length.

**Figure 7.**
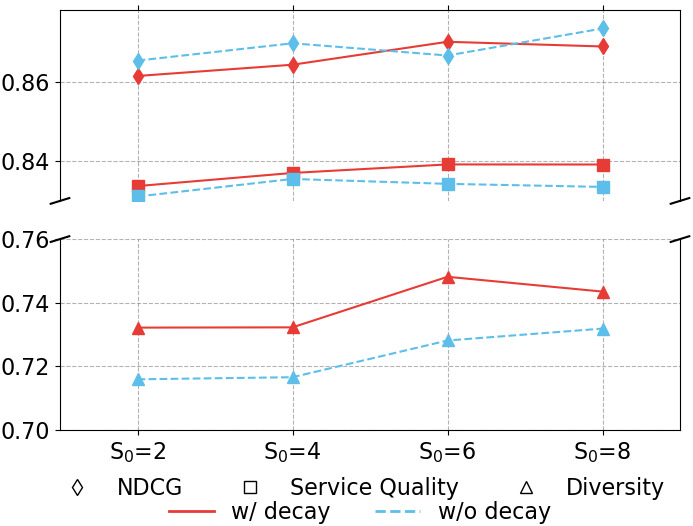
The mean objective values of dynamic graph–based bacteria colony optimization for multiobjective physician recommendation (DyGMO-PR) with different *S*_0_ settings. NDCG: normalized discounted cumulative gain.

**Figure 8.**
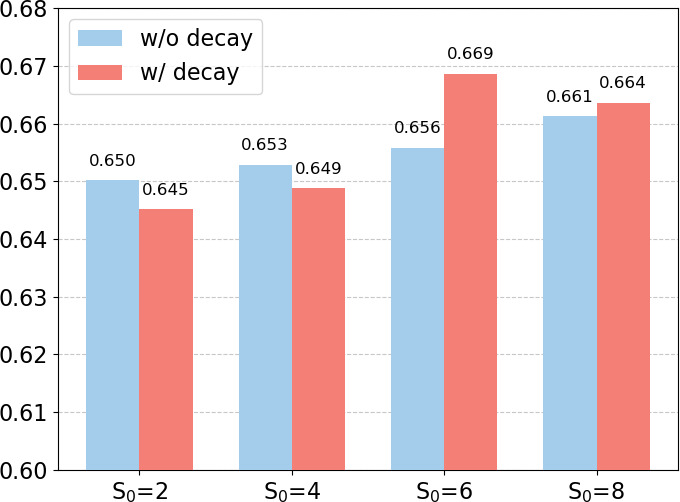
The hypervolume values of dynamic graph–based bacteria colony optimization for multiobjective physician recommendation (DyGMO-PR) with different *S*_0_ settings.

Figure 7 and Figure 8 depict the performance of DyGMO-PR with different step size settings. The step size decay strategy in Equation 9 is compared with an alternative strategy in which the step size remains constant. As shown in Figure 7, our proposed decay strategy outperforms the alternatives in terms of service quality and diversity performance. The hypervolume comparison results in Figure 8 show that the constant step size strategy performs better when the initial step size is small (ie, *S*_0_=2 and *S*_0_=4), whereas our decay strategy is more effective for a larger initial step size (ie, *S*_0_=6 and *S*_0_=8). Notably, when the *S*_0_ is set to 6, our decay strategy achieves the best overall performance. These results indicate that the proposed step size schedule facilitates rapid convergence in the early stages of the optimization process while enabling fine-tuning in later stages.

### Case Studies

To assess the interpretability of DyGMO-PR, we randomly selected a patient for case studies. The analysis first examines the physician recommendation lists, then investigates the evolution of the physician graph.

The focal patient for the case studies is a premature infant with twelve online consultation records on the online health care consultation platform. This patient has consulted 8 different physicians, with one physician (ID: 14) being consulted 4 times. The consultations spanned multiple departments, including pediatrics, rehabilitation, gastroenterology, otolaryngology, dermatology, and others.

As shown in Multimedia Appendix 3, the recommendations generated by DyGMO-PR outperform those produced by the benchmark methods across all 3 recommendation objectives. To provide a deeper insight, we randomly selected 2 recommended physician lists from the Pareto front obtained by DyGMO-PR (see Multimedia Appendix 3). Recommendation list 1 exhibits optimal accuracy performance with lower diversity of physician expertise (accuracy=0.944, service quality=0.793, and diversity=0.489). In this list, the diseases that the other 6 physicians specialize in treating are closely related to those that can be treated by Physician ID 14. In contrast, recommendation list 2 demonstrates a more balanced performance across the 3 recommendation objectives, including accuracy (0.788), service quality (0.814), and diversity (0.625). Here, 3 of the recommended physicians have been consulted previously, with Physician ID 14 once again ranked first. The remaining physicians specialize in a broader and less directly related range of diseases compared to those in recommendation list 1, clearly illustrating the inherent trade-off between recommendation accuracy and diversity of physician expertise.

The dynamic graph learning strategy updates the graph structure to better align with the recommendation objectives. We further examined the evolution of the physician graph during the optimization process. Multimedia Appendix 4 presents the properties of the initial and converged graph for this patient. Although the number of nodes remains constant, the number of edges increases substantially, with the average node degree rising by 27.145 and graph density increasing by 0.009.

To investigate graph local structural changes, we randomly selected 2 physician nodes (ID 25 and ID 971) and analyzed their connections in both the initial and converged physician graphs. Physician ID 25 specializes in treating neonatal jaundice, pneumonia, and pediatric enteritis, while Physician ID 971 focuses on anorexia, asthma, recurrent respiratory infections, common cold, cough, precocious puberty, and indigestion. As detailed in Multimedia Appendix 5, Physician ID 25 gains 8 new neighbors in the converged graph, and the diseases treated by these new neighbors are closely aligned with the patient’s historical consultations. For Physician ID 971, 5 initial neighboring physicians are removed, and 3 new neighbors are added. The newly connected physicians specialize in a broader range of diseases than those removed, which not only aligns more closely with the patient’s historical conditions but also enhances the diversity of diseases covered in the recommendation list.

Moreover, we analyzed the structural changes of the entire physician graph before and after evolution (detailed results are provided in Multimedia Appendix 6). In the initial graph, all physicians had equal in-degree and out-degree. After evolution, we stratified physicians by their in-degree/out-degree ratio, which reflects a physician’s relative attractiveness as a target in the evolved directed graph. The results show that physicians ranked in the top 50 by this ratio achieved substantially higher accuracy, service quality, and diversity of physician expertise compared to other groups. Among the 8 physicians the patient had actually consulted, 7 exhibited an in-degree/out-degree ratio well above 1. This finding corroborates that the graph evolution enhances the prominence of physicians relevant to the patient’s historical conditions. This indicates that the graph evolution process effectively reshapes the network structure to improve recommendation performance. These findings provide global-level interpretability and validate the effectiveness of the proposed dynamic graph learning mechanism.

The above case studies demonstrate the effectiveness and interpretability of the DyGMO-PR algorithm. The analysis of 2 selected lists from its Pareto front illustrates the algorithm’s ability to produce diverse, high-quality options that balance the patient’s need for accuracy, service quality, and disease coverage. Furthermore, the physician graph analysis reveals that the dynamic graph evolution actively reshapes the relationships between physicians. Adding and removing directed connections enables the heuristic searching process to uncover specialists whose expertise aligns more closely with the patient’s personalized needs. The resulting graph provides an interpretable map of why certain physicians are recommended, thereby improving the transparency and trustworthiness of the system.

## Discussion

Physician recommender systems in online health care consultation assist patients in navigating the challenge of selecting a physician from an overwhelming number of available options. In this research, we proposed a novel DyGMO-PR. DyGMO-PR simultaneously optimizes the recommendation accuracy, physician service quality, and the diversity of physician expertise. This new method introduces a dynamic physician relationship learning mechanism that mitigates data sparsity and stabilizes the search process for recommendation lists.

The contributions of this research can be summarized in 3 main aspects. First, we propose a multiobjective physician recommendation framework grounded in BCO. Unlike conventional methods that optimize a single objective, we define 3 objectives that reflect patients’ key concerns: accuracy, service quality, and diversity of physician expertise. DyGMO-PR effectively balances these objectives and can be readily extended to other multiobjective recommendation scenarios by customizing the objective functions. Second, we propose a physician graph-based encoding scheme that assigns virtual scores to quantify each physician’s potential contribution. This design enhances the stability and convergence of heuristic optimization in sparse and discrete solution spaces. Third, we propose a dynamic physician graph evolution mechanism that learns new physician relationships and refines the physician graph to support interpretable recommendations.

The proposed method offers substantial practical value for online health care platforms, physicians, and patients. For platforms, DyGMO-PR provides a flexible and tunable foundation for physician recommendation. By generating a Pareto-optimal set of solutions, platforms can assign different weights to the 3 objectives according to user needs. For instance, they may prioritize accuracy for new users, emphasize service quality for premium services, or enhance diversity for users with unclear symptoms. This adaptability allows platforms to tailor recommendations according to user characteristics or platform development stages. For physicians, the method increases the visibility of high-quality specialists who might otherwise be overlooked due to sparse interaction data. For patients, DyGMO-PR delivers personalized and interpretable recommendation lists that balance accuracy, service quality, and disease coverage. This is particularly valuable for patients with limited medical knowledge or complex conditions, as it broadens their access to relevant and trustworthy specialists.

The DyGMO-PR framework is designed to be generalizable to other domains involving multiobjective recommendation in the presence of sparse interaction data. The method requires three types of input data: (1) user-item interaction history, (2) item attribute information, and (3) a relational structure among items. These data requirements are commonly met across various real-world scenarios. Within the health care domain, beyond physician recommendation, the method can be applied to hospital referral recommendation, where patients are matched to hospitals based on specialty strength and service quality. Beyond health care, the proposed approach is also well-suited for e-commerce product recommendation (eg, balancing relevance, product ratings, and category diversity) and content platform recommendation (eg, news or videos, balancing content matching, quality, and topic diversity). In each case, the approach can be adapted by redefining the optimization objectives and constructing domain-specific relational graphs.

There are several promising directions for future research. First, DyGMO-PR could be extended to address other real-world problems that require identifying multiple optimal solutions. Second, the accuracy objective relies on patients’ historical consultation records as ground truth. However, these records may contain noise because some patients with limited medical knowledge do not always choose the most appropriate physician. We mitigated this noise by retaining only patients with at least 5 consultations and using XSimGCL, a robust contrastive learning model for rating prediction. Nonetheless, some noise inevitably remains, and future research could incorporate more precise feedback signals (eg, postconsultation satisfaction ratings) or external medical knowledge to eliminate noise and improve rating accuracy. Third, incorporating deeper medical domain knowledge, such as disease comorbidity relationships or symptom-disease co-occurrence networks, may further enhance the reliability of the physician graph and improve recommendation performance. Such external knowledge could provide a more robust foundation for dynamic graph learning. We hope this study will inspire future research in optimization-based multiobjective recommender systems.

## Supplementary material

10.2196/88854 Multimedia Appendix 1 Detailed pseudo code of physician graph walking–based bacteria chemotaxis.

10.2196/88854 Multimedia Appendix 2 Detailed parameter setting of candidate algorithms.

10.2196/88854 Multimedia Appendix 3 Detailed 3D illustration of Pareto front obtained by algorithms for different K values.

10.2196/88854 Multimedia Appendix 4 Detailed properties of initial and converged physician graphs.

10.2196/88854 Multimedia Appendix 5 Detailed statistical summaries and structural representations of the local graphs surrounding physician ID 25 and physician ID 971.

10.2196/88854 Multimedia Appendix 6 Detailed structural changes of the entire physician graph before and after evolution.

## Abbreviations

BCO: bacterial colony optimization
BERT: Bidirectional Encoder Representations from Transformers
DCG: discounted cumulative gain
DyGMO-PR: dynamic graph–based bacteria colony optimization for multiobjective physician recommendation
IDCG: ideal discounted cumulative gain
MOEA/D: multiobjective evolutionary algorithm based on decomposition
MOFA: multiobjective firefly algorithm
MOPSO: multiobjective particle swarm optimization
NDCG: normalized discounted cumulative gain
NSGA-II: nondominated sorting genetic algorithm II
RQ: research question
SPEA-2: strength Pareto evolutionary algorithm 2
XSimGCL: extremely simple graph contrastive learning
