# Supplementary material for: A Dynamic Graph–Based Multiobjective Optimization Method for Physician Recommendation: Development and Evaluation Study
Source: JMIR Med Inform. 2026 Jul 31;14:e88854. doi: 10.2196/88854 (PMC13430641; doi:10.2196/88854)
Supplement: Multimedia Appendix 1 [file medinform-v14-e88854-s001.docx]

**Multimedia Appendix 1**

| **Pseudo code: Physician graph walking based bacteria chemotaxis** |
| --- |
| **Input:** physician graph: $G_{p}$, graph walk step size num of current iteration: ${stepsize}_{t}$, bacteria population: $B$, current user: $u$  **Output:** updated bacteria population after graph walking: $B^{'}$  For each bacteria $b_{j}\in B$:  For each walk $step=1$ to ${stepsize}_{t}$:  Select a physician $p_{i}$ from the physician list of $b_{j}$ according to Eq. (6)  Create a physician set ${Phy}_{candidate}$ composed of neighbor physicians connected to $p_{i}$  Select an objective from the three objectives randomly with equal probability as $obj$  For each neighbor physician $p_{can}\in{Phy}_{candidate}$:  Compute the objective gain ${gain}_{can}$ using Eq. (7) and Eq. (8)  If ${gain}_{can}<0$:  Remove the neighbor physician from ${Phy}_{candidate}$  Select a $p_{can}^{'}$ to walk to from ${Phy}_{candidate}$ with a probability proportional to its ${gain}_{can}$  Replace $p_{i}$ with $p_{can}^{'}$, and update $b_{j}$ to $b_{j}^{'}$  Update $B$ by replacing $b_{j}$ with $b_{j}^{'}$ |
