# Supplementary material for: A Dynamic Graph–Based Multiobjective Optimization Method for Physician Recommendation: Development and Evaluation Study
Source: JMIR Med Inform. 2026 Jul 31;14:e88854. doi: 10.2196/88854 (PMC13430641; doi:10.2196/88854)
Supplement: Multimedia Appendix 2 [file medinform-v14-e88854-s002.docx]

**Multimedia Appendix 2**

**Table A2-1. Parameter setting of candidate algorithms.**

| Algorithm | Parameter | Meaning | Value |
| --- | --- | --- | --- |
| NSGA2 | *p_c_* | Probability of crossover | 0.8 |
|  | *p_m_* | Probability of mutation | 0.1 |
|  | *T* | Maximum iterations | 100 |
|  | *N* | Population size | 100 |
|  | *N_rep_* | External document size | 100 |
| MOPSO | *m* | The number of divisions in each objective | 10 |
|  | *w* | Initial Inertia Weight Factor | 0.4 |
|  | *T* | Maximum iterations | 100 |
|  | *N* | Population size | 100 |
|  | *N_rep_* | External document size | 100 |
| MOFA | *α* | Randomization parameter | 0.1 |
|  | *β_0_* | Attractiveness of firefly | 1 |
|  | *γ* | Light Absorption coefficient | 1 |
|  | *T* | Maximum iterations | 100 |
|  | *N* | Population size | 100 |
|  | *N_rep_* | External document size | 100 |
| SPEA2 | *p_c_* | Probability of crossover | 0.8 |
|  | *p_m_* | Probability of mutation | 0.1 |
|  | *T* | Maximum iterations | 100 |
|  | *N* | Population size | 100 |
|  | *N_rep_* | External document size | 100 |
| MOEA/D | *p_c_* | Probability of crossover | 0.8 |
|  | *p_m_* | Probability of mutation | 0.1 |
|  | *n_s_* | Number of nearest neighbor individuals | 10 |
|  | *T* | Maximum iterations | 100 |
|  | *N* | Population size | 100 |
|  | *N_rep_* | External document size | 100 |
| TRMOBCO | *N_s_* | Run and tumbling steps | 4 |
|  | *PED^0^* | Initial population energy degree | 1 |
|  | *p_0_* | Standard dissipation probability | 1 |
|  | *T* | Maximum iterations | 100 |
|  | *N* | Population size | 100 |
|  | *N_rep_* | External document size | 100 |
| DyGMO-PR | *N_s_* | Run and tumbling steps | 4 |
|  | *S_0_* | Initial random walk step size | 6 |
|  | *PED^0^* | Initial population energy degree | 1 |
|  | *p_0_* | Standard dissipation probability | 1 |
|  | *T* | Maximum iterations | 100 |
|  | *N* | Population size | 100 |
|  | *N_rep_* | External document size | 100 |
