# Supplementary material for: A Dynamic Graph–Based Multiobjective Optimization Method for Physician Recommendation: Development and Evaluation Study
Source: JMIR Med Inform. 2026 Jul 31;14:e88854. doi: 10.2196/88854 (PMC13430641; doi:10.2196/88854)
Supplement: Multimedia Appendix 3 [file medinform-v14-e88854-s003.docx]

**Multimedia Appendix 3**

**Figure A3-1. 3-D illustration of Pareto front obtained by algorithms for different** $K$**values.**

| 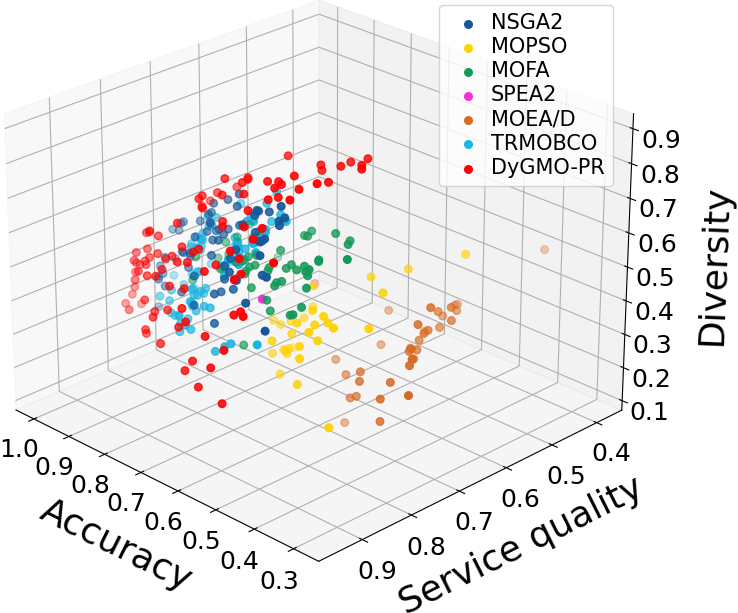 | 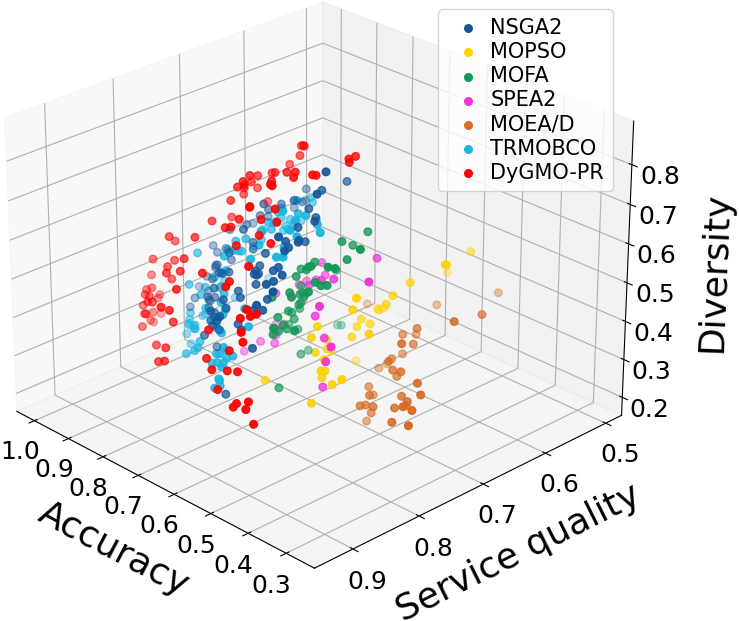 |
| --- | --- |
| (a) $K=6$ | (b) $K=8$ |
| 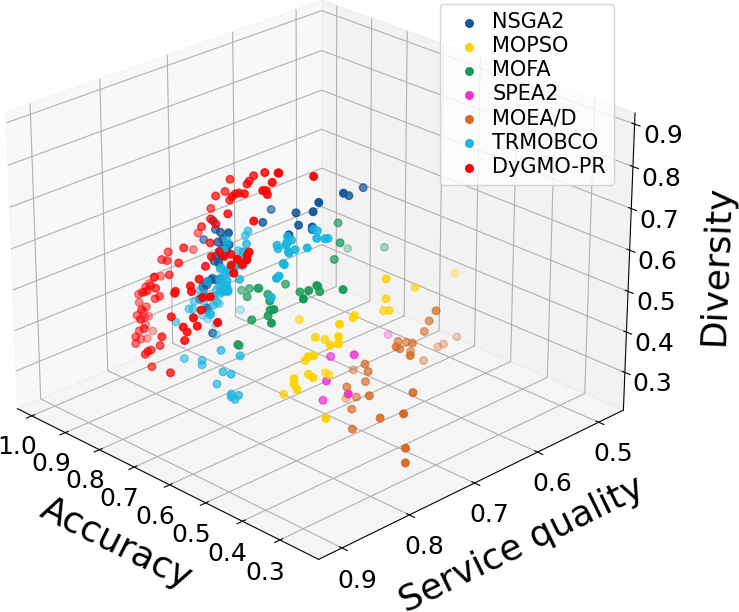 | 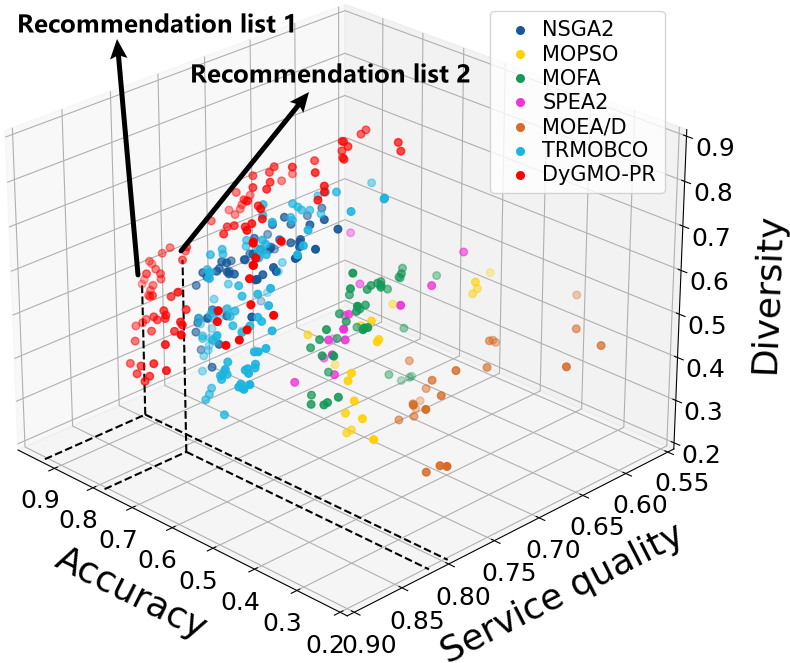 |
| (c) $K=10$ | (d) $K=12$ |
| 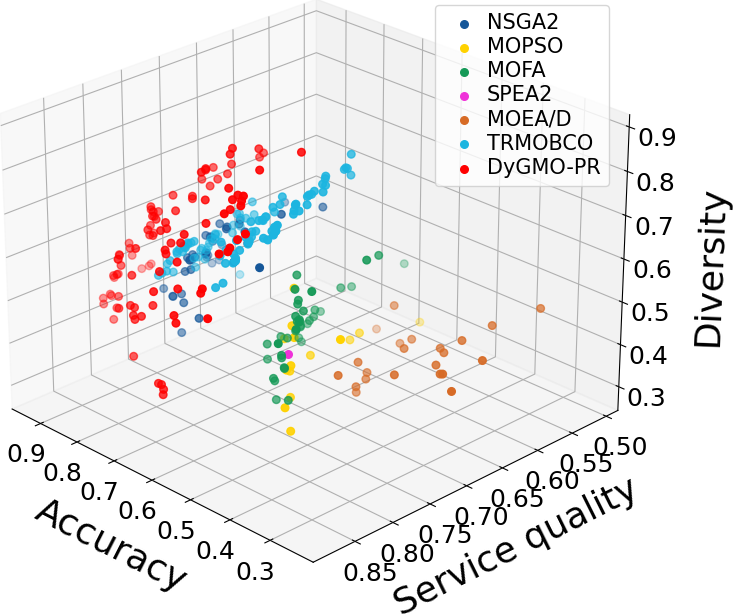 | 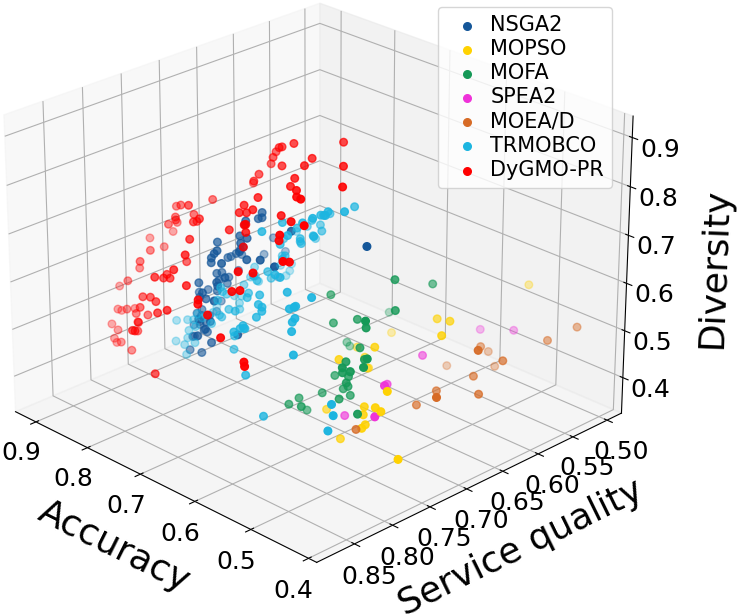 |
| (e) $K=14$ | (f) $K=16$ |
| 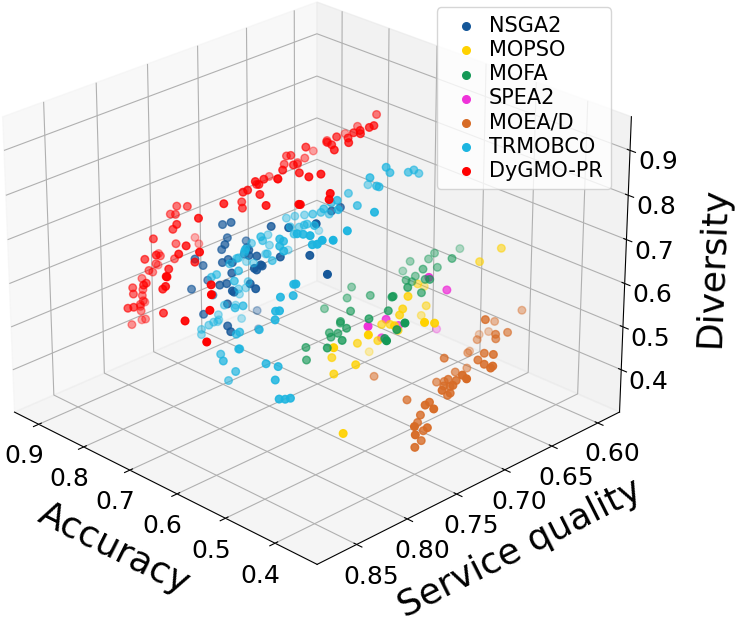 | 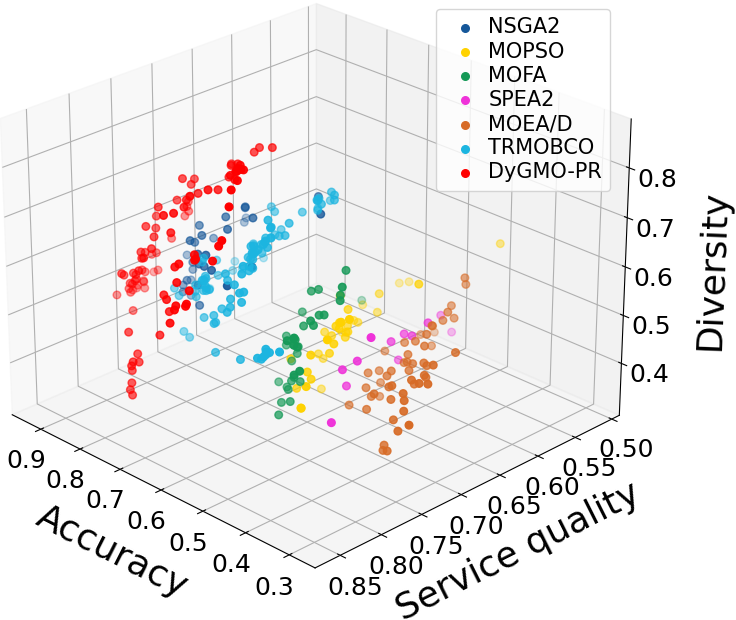 |
| (g) $K=18$ | (h) $K=20$ |
