# Supplementary material for: A Dynamic Graph–Based Multiobjective Optimization Method for Physician Recommendation: Development and Evaluation Study
Source: JMIR Med Inform. 2026 Jul 31;14:e88854. doi: 10.2196/88854 (PMC13430641; doi:10.2196/88854)
Supplement: Multimedia Appendix 4 [file medinform-v14-e88854-s004.docx]

**Multimedia Appendix 4**

**Table A4-1. Properties of initial and converged physician graphs.**

|  | Number of Nodes | Number of Edges | Average Degree | Graph Density |
| --- | --- | --- | --- | --- |
| Initial graph | 1377 | 107136 | 155.608 | 0.057 |
| Converged graph | 1377 | 125825 | 182.752 | 0.066 |
