# Supplementary material for: A Dynamic Graph–Based Multiobjective Optimization Method for Physician Recommendation: Development and Evaluation Study
Source: JMIR Med Inform. 2026 Jul 31;14:e88854. doi: 10.2196/88854 (PMC13430641; doi:10.2196/88854)
Supplement: Multimedia Appendix 5 [file medinform-v14-e88854-s005.docx]

**Multimedia Appendix 5**

**Table A5-1. Statistics about graph around physician no.25 and no.971.**

| Characteristic | Graph of physician ID 25 | | Graph of physician ID 971 | |
| --- | --- | --- | --- | --- |
|  | Before | After | Before | After |
| No. of neighbors | 34 | 42 | 110 | 108 |
| No. of edges | 936 | 1055 | 5652 | 5323 |
| Avg. degree | 55.059 | 50.238 | 102.764 | 98.574 |
| Graph density | 0.834 | 0.613 | 0.471 | 0.461 |
| Avg. patient rating | 0.531 | 0.593 | 0.527 | 0.545 |
| Avg. service quality | 0.614 | 0.648 | 0.607 | 0.618 |
| Avg. no. of skilled disease | 9.212 | 9.122 | 8.752 | 9.103 |

**Figure A5-1. Local graph structure of Physician ID 25 and Physician ID 971.**

*Note: Red node: Physicians ID 25 and ID 971. Yellow node: initial connections that were latterly removed. Green node: new connections added. Blue node: other physicians in the graph. Red lines: edges between Physicians ID 25 and ID 971 and their neighbors. Gray lines: edges between other physicians.*

| 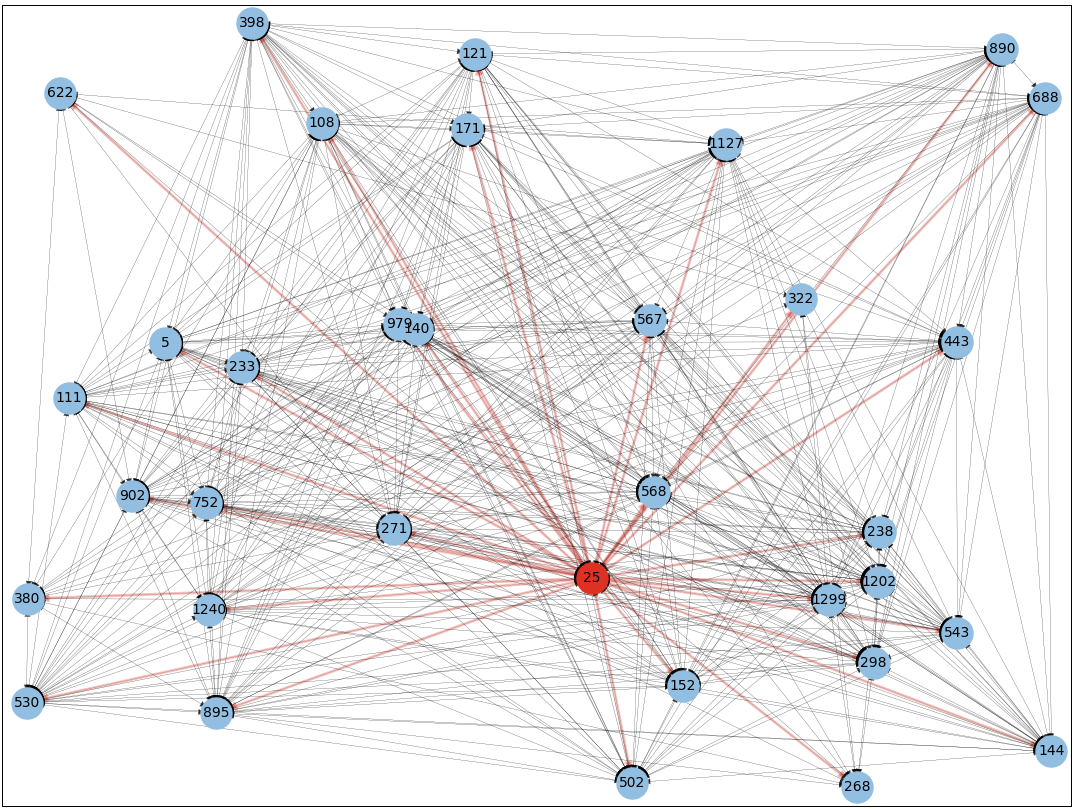 | 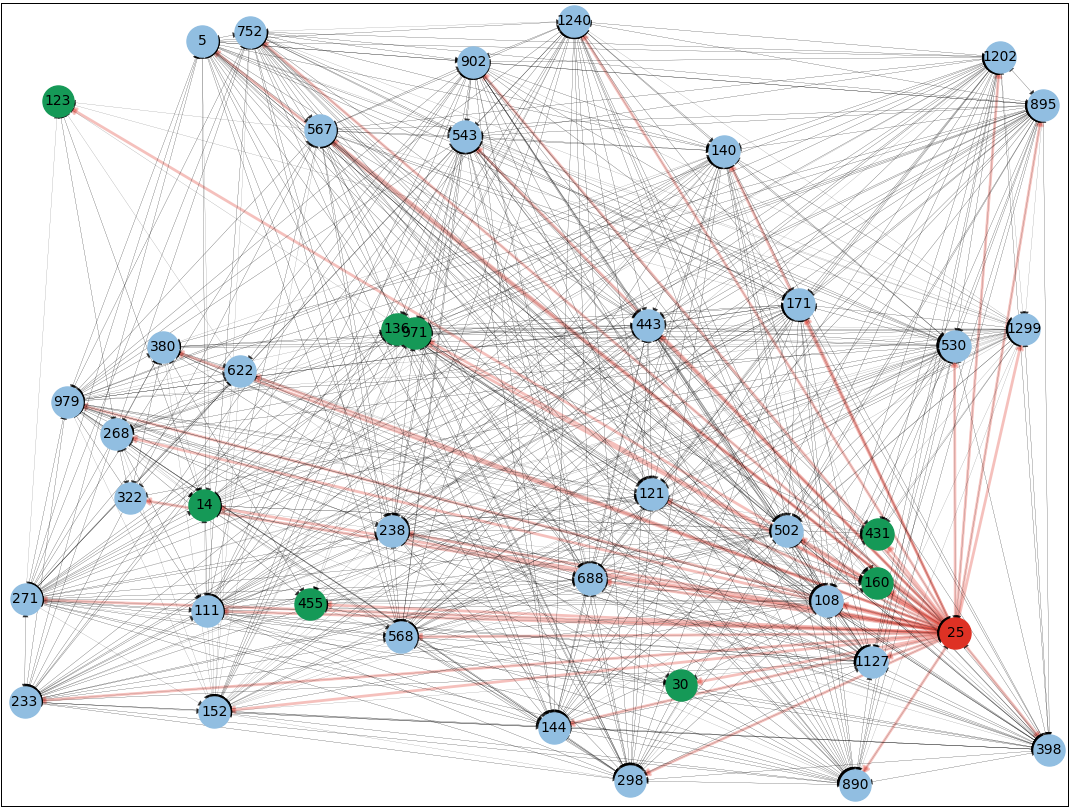 |
| --- | --- |
| (a) Initial local graph for Physician ID 25 | (b) Final local graph for Physician ID 25 |
| 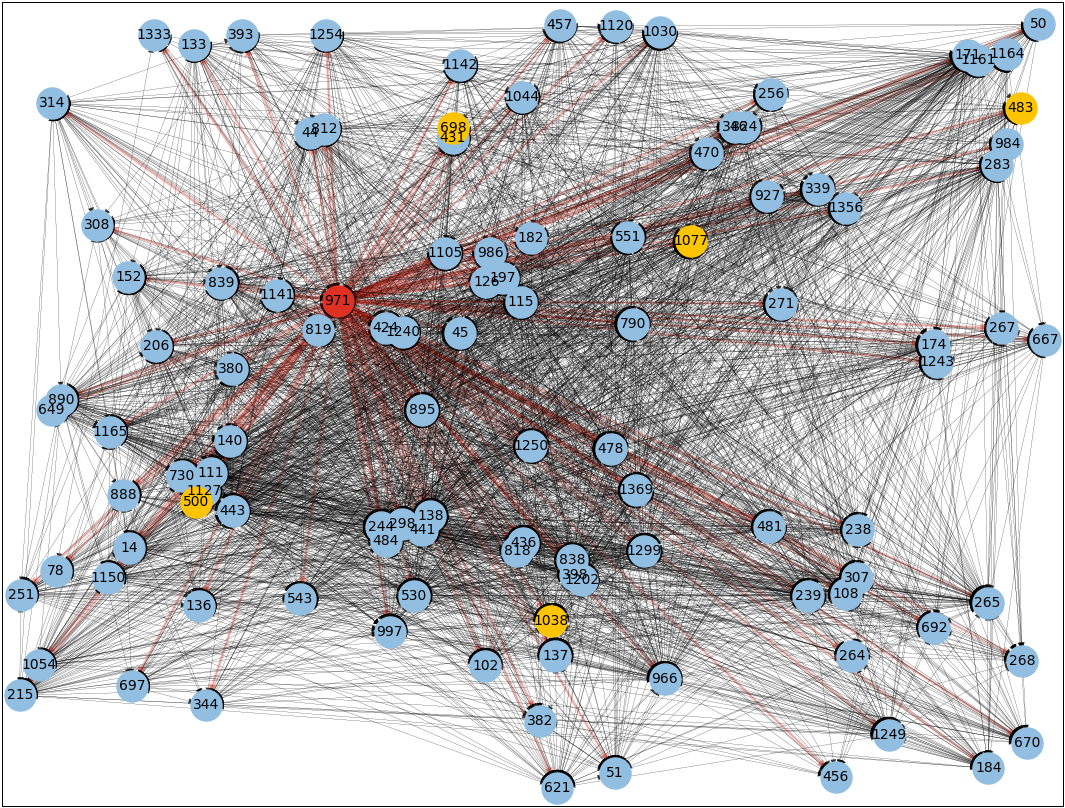 | 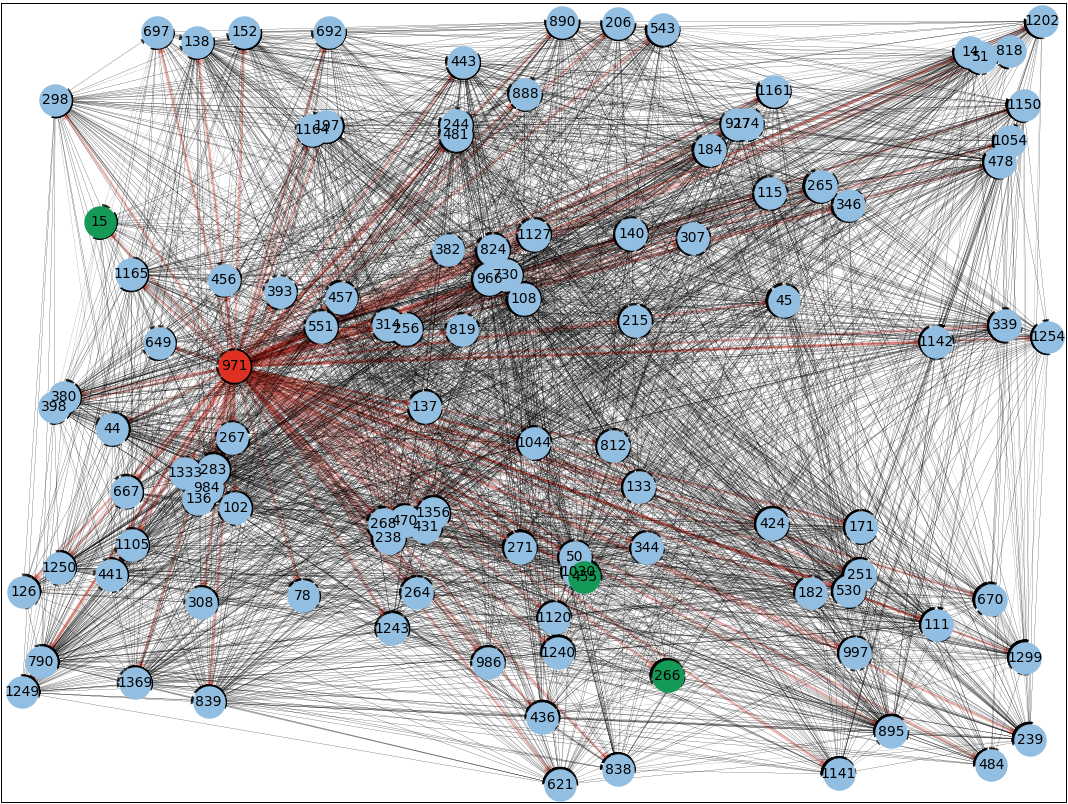 |
| (c) Initial local graph for Physician ID 971 | (d) Final local graph for Physician ID 971 |

**Table A5-2. Professionalized disease of the eight physicians consulted by the case patient.**

| Physician index | Professionalized diseases |
| --- | --- |
| 136 | Tonsillitis, Hand-foot-and-mouth disease, Bronchitis, Indigestion, Bronchopneumonia, Neonatal pulmonary hypertension |
| 429 | Children's short stature |
| 14 | Constipation, Lactose intolerance, Gastroesophageal reflux disease, Food allergy, Liver dysfunction, Indigestion, Diarrhea, Helicobacter pylori infection, Jaundice, Anorexia |
| 430 | Post-mature infant, Diarrhea, Cytomegalovirus disease, Neonatal pathological jaundice, Viral rash, Respiratory infections, Gastrointestinal dysfunction, Respiratory syncytial virus pneumonia, Premature infant |
| 431 | Autism, Developmental delay, Cerebral palsy, Chronic rhinitis, Spinal cord injury, Cerebrovascular disease, Anorexia |
| 266 | Otitis media, Auricular deformities, Deafness, Sinusitis, Adenoid hypertrophy, Sleep apnea syndrome, Allergic rhinitis |
| 15 | Eczema, Urticaria, Pigmented nevi, Dry eczema, Perforating folliculitis and follicular keratosis, Eczematous dermatitis, Flat warts, Infantile hemangioma, Ota nevus |
| 160 | Skin disease, Dermatitis, Urticaria, Eczema, Fungal skin disease, Freckles, Folliculitis, Acne, Fungal infection, Viral warts, Scars |

**Table A5-3. Professionalized diseases of new physician neighbors for physician no. 25.**

| Physician index | Professionalized diseases |
| --- | --- |
| 14 | Constipation, Lactose intolerance, Gastroesophageal reflux disease, Food allergy, Liver dysfunction, Indigestion, Diarrhea, Helicobacter pylori infection, Jaundice, Anorexia |
| 30 | Birthmarks, Wrinkles, Skin laxity, Nevi, Stretch marks, Pigmentation spots, Acne, Body odor, Skin tumors |
| 123 | Food allergy, Atopic dermatitis, Milk protein allergy, Allergic rhinitis, Allergic purpura |
| 136 | Tonsillitis, Hand-foot-and-mouth disease, Bronchitis, Indigestion, Bronchopneumonia, Neonatal pulmonary hypertension |
| 160 | Skin disease, Dermatitis, Urticaria, Eczema, Fungal skin disease, Freckles, Folliculitis, Acne, Fungal infection, Viral warts, Scars |
| 431 | Autism, Developmental delay, Cerebral palsy, Chronic rhinitis, Spinal cord injury, Cerebrovascular disease, Anorexia |
| 455 | Eczema, Skin disease, Urticaria, Dermatitis, Café-au-lait spots, Pigmented skin disease, Hemangioma, Port-wine stain, Ota nevus, Acne, Vitiligo, Alopecia areata, Tinea capitis, Molluscum contagiosum, Warts |
| 971 | Anorexia, Asthma, Recurrent respiratory infections, Cold, Cough, Precocious puberty, Indigestion |

**Table A5-4. Professionalized diseases of new physician neighbors for physician no. 971**

| Physician index | Professionalized diseases |
| --- | --- |
| 15 | Eczema, Urticaria, Pigmented nevi, Dry eczema, Perforating folliculitis and follicular keratosis, Eczematous dermatitis, Flat warts, Infantile hemangioma, Ota nevus |
| 266 | Otitis media, Auricular deformities, Deafness, Sinusitis, Adenoid hypertrophy, Sleep apnea syndrome, Allergic rhinitis |
| 455 | Eczema, Skin disease, Urticaria, Dermatitis, Café-au-lait spots, Pigmented skin disease, Hemangioma, Port-wine stain, Ota nevus, Acne, Vitiligo, Alopecia areata, Tinea capitis, Molluscum contagiosum, Warts |

**Table A5-5. Professionalized diseases of disconnected physician neighbors for physician no. 971**

| Physician index | Professionalized diseases |
| --- | --- |
| 483 | Hypertension, Cold |
| 500 | Headache, Cold |
| 698 | Migraine, Dizziness, Headache, Weakness, Tremor, Convulsion |
| 1038 | Cold |
| 1077 | Cough, Pulmonary nodules, Snoring, Respiratory tract infection |
