# Supplementary material for: A Dynamic Graph–Based Multiobjective Optimization Method for Physician Recommendation: Development and Evaluation Study
Source: JMIR Med Inform. 2026 Jul 31;14:e88854. doi: 10.2196/88854 (PMC13430641; doi:10.2196/88854)
Supplement: Multimedia Appendix 6 [file medinform-v14-e88854-s006.docx]

**Multimedia Appendix 6**

**Table A6-1. Statistics about global graph before evolution.**

| Physician Group | In-degree/out-degree ratio | Avg. patient rating | Avg. service quality | Avg. no. of skilled disease |
| --- | --- | --- | --- | --- |
| 1-50 | 1 | 0.477 | 0.616 | 13.520 |
| 51-200 | 1 | 0.479 | 0.613 | 10.180 |
| 201-500 | 1 | 0.466 | 0.591 | 7.557 |
| 501-1377 | 1 | 0.477 | 0.603 | 5.569 |

**Note:** The first column groups physicians by their ranking based on initial degree, where 1-50 denotes the top 50 physicians.

**Table A6-2. Statistics about global graph after evolution.**

| Physician Group | In-degree/out-degree ratio | Avg. patient rating | Avg. service quality | Avg. no. of skilled disease |
| --- | --- | --- | --- | --- |
| 1-50 | 8.068 | 0.687 | 0.809 | 11.380 |
| 51-200 | 1.441 | 0.534 | 0.769 | 10.487 |
| 201-500 | 0.987 | 0.491 | 0.652 | 9.197 |
| 501-1377 | 0.696 | 0.447 | 0.544 | 5.078 |

**Note:** The first column groups physicians by their ranking based on initial degree, where 1-50 denotes the top 50 physicians.

**Table A6-3. In-degree/out-degree ratios of the case patient's consulted physicians before and after graph evolution.**

| Physician ID | In-degree/out-degree ratio before evolution | In-degree/out-degree ratio after evolution | Rank of in-degree/out-degree ratio after evolution (total physician num=1377) |
| --- | --- | --- | --- |
| 136 | 1 | 3.250 | 32 |
| 429 | 1 | 1.000 | 312 |
| 14 | 1 | 14.191 | 5 |
| 430 | 1 | 11.565 | 8 |
| 431 | 1 | 3.679 | 27 |
| 266 | 1 | 13.395 | 7 |
| 15 | 1 | 13.707 | 6 |
| 160 | 1 | 9.212 | 10 |
